# Exclusive use of digital PCR allows an absolute assay of heat-killed *Lactobacilli* in foods targeting multiple copies of 16S rDNA

**DOI:** 10.1038/s41598-020-69206-5

**Published:** 2020-07-29

**Authors:** Takashi Soejima, Miyuki Tanaka, Koji Yamauchi, Fumiaki Abe

**Affiliations:** Functional Food Ingredients Group, Food Ingredients and Technology Institute, R & D Division, Morinaga Milk Industry Co. Ltd., 5-1-83, Higashihara, Zama, Kanagawa 252-8583 Japan

**Keywords:** Assay systems, Genetic testing

## Abstract

The real-time PCR (qPCR) and digital PCR (dPCR) to amplify a single-copy of house-keeping genes (i.e., *hsp60, pheS* or *tuf*) are used for the assay of limited microbial species. In general, with a single-copy gene, there are obviously varied DNA sequences for even the same microbial species, which could cause difficulties with design of primers and probes for PCR when targeting various single copy genes. In general, for identification by dPCR (as a representative case: *Lactobacillus paracasei*), accumulated DNA sequence information of 16S rDNA, which is much more frequently used, should be targeted. In contrast, next-generation sequencing revealed that there are five copies of 16S rDNA in a live *L. paracasei* MCC1849. Therefore, we aimed to reveal, if heat-killed *L. paracasei* supplemented in nutritional foods that aid the host immune system have the relevant five copies per chromosomal DNA, and if the relevant copies remain unchanged on the same chromosomal DNA or remain to be different chromosomal DNA fragments. So, we revealed the actual distribution of the potential original five copies of 16S rDNA using our innovative dPCR, in which both 16S rDNA and *hsp60* genes were simultaneously elongated. The molecular ratios of 16S rDNA/*hsp60* dispersed in the dPCR chip were then estimated. The 16S rDNA/*hsp60* molecular ratios of the heat-killed *L. paracasei* in foods, resultantly ranged from 5.0 to 7.2, being the same or higher than that of the five copies determined by next-generation sequencing. The 16S rDNA copy number/ratio indicated the chromosomal DNA molecular number and the associated cell number. As significance, different nutritional foods could potentially cause the loss of chromosomal DNA of supplemented beneficial microbes to a much greater degree. Our absolute dPCR does not require standard correlative samples for the estimation of final products. The estimation principle of the ratio of 16S rDNA/a house-keeping single-copy gene by our absolute dPCR could lead to a useful and accurate assay for various nutritional foods.

## Introduction

The specific term, digital polymerase chain reaction (dPCR) first appeared in a report by Kinzler and Vogelstein^1^. Since then, dPCR has been applied to viable cells or active viruses in test samples in the fields of clinical, environmental, and food science^2–11^. Especially, with respect to good bacteria in the food science, recent reports have implied they are not only merely viable, but they also could have beneficial effects by mediating immunomodulation, such as the heat-killed *Lactobacilli* (*Lactobacillus paracasei* and *Enterococcus faecalis*)^12,13^. Thus, relevant research has targeted the immunogenic components of heat-killed *Lactobacilli* using mouse and human immune cells to reveal that ssRNA (23S rRNA and 16S rRNA) and chromosomal DNA could greatly increase host immune response by mediating the relevant immune cells^12,14,15^. In contrast, there is reported microbial peptidoglycan (PGN) to induce innate immunity of host mammalian cells, and the PGN actually interacts with macrophages and lymphocytes of the immune system^16,17^. Macrophage activation accelerates producing cytokines responsible for clinical effects to pathogenic infections^18,19^. Actually, it was demonstrated the intact and/or close to intact form of *Lactobacilli* that sustains both the relevant genetic components and PGN could be recommendable to increase host immune response^13^. Also, in view of originally food preservability it is taken for granted that the heat-killed good germs is more excellent than the associated live cells owing to not-growth of the relevant cells during food storage. However, the supplementation of heat-killed pathogenic bacteria to foods for activation of human immune response should never been examined. If the live form of the relevant were in part sustained during the production of heat-killed pathogenic bacteria, their supplemented foods could cause food poisoning at a worst case.

So, some nutritional foods (abbreviated as NFs; a singular form of NF) supplemented with heat-killed *Lactobacilli* that comprises of both the relevant genetic components and PGN becomes launched in the worldwide market. In relation to B2B business, a potentially outsider company that intend to purchase powders of heat-killed *L. paracasei* demands us (supplier) accurate number of the heat-killed relevant cells per 1 g of powders for a smoothly commercial transaction. Furthermore, its outsider company would like to newly add our powder of heat-killed *L. paracasei* to originally producing foods and NFs of its outsider company. Hence, the establishment of an accurate assay to analyse close to intact form of heat-killed *L. parcasei*-supplemented NFs is needed. And, also from the viewpoint of supplier of us, to guarantee i.e. 1.0 × 10^8^ cells/g of NFs products, we need to circumvent the supplementation of relevant cells higher by tenfold to cause too much production cost.

In the accurate traditional dPCR assay, a gradually increasing concentration of purified DNA is applied by using 10-, 8-, 6-, 4-, 2-fold dilution or no dilution, leading to a parallel increase in dPCR raw data (copies/µl of the dPCR master mix)^20,21^. In contrast, in the traditional real-time PCR (qPCR), an evidently increased concentration of DNA template at a logarithmic scale (by tenfold) can contribute to a significant increase in the Ct value.

dPCR can precisely distinguish different concentrations of the applied DNA^22^. However, we wanted to assay a specific *Lactobacilli* cell rather than a gene (DNA) in bio-material and nutritional foods. Nonetheless, dPCR typically focuses on the assay of a target gene through the amplification of purified DNA extracted from various test samples^2,6,7,10,11^. Even if the test samples contain the same number of targeted cells, the significant variance in the DNA recovery rate during the DNA extraction could trigger a lower output, between two- and fourfold, than the original concentration of cells^20,21^. We believe that the assay should be optimised to stay unaffected even by different DNA recovery rates in the same sample.

The dPCR machine was originally developed for absolute quantification without any correlative standards; nonetheless, the assay was performed using the logarithmic scale of a correlative target-gene-standard curve for various test samples^8,23–25^. So, performing an absolute (without any standard curves) assay of targeted cells by dPCR alone for nutritional foods is challenging.

Indeed, so far, a single-copy gene on one chromosomal DNA has been targeted to specifically assay cells in dPCR; thus, the raw data obtained (gene copy number) should be theoretically identical to the number of chromosomal DNA molecules, and in extension, the targeted microbial cells. In general, the use of the 16S rRNA gene (16S rDNA) is much more frequent and has a much broader impact than the use of a single-copy gene such as *hsp60*, *pheS*, *rpoB*, or *tuf* in identifying microorganisms^26,27^. When using 16S rDNA for the accurate identification of the *L. paracasei* MCC1849 strain (NITE BP-01633), we need to pay attention to five copies of 16S rDNA coded on one chromosomal DNA of the relevant live cells according to the previously done commission analysis with TaKaRa-Bio (Kusatsu, Japan) next-generation sequencing (NGS). Consequently, in dPCR assays, it is important to accurately evaluate the physical distribution of the five copies of 16S rDNA into each well of a dPCR chip. Additionally, in comparing heat-killed (HK) *L. paracasei* previously subjected to 90 °C for 15 min and additional sterilisation during the production of NF with the relevant live cells, the degree of DNA fragmentation (degree of physical decomposition) in the former, HK*-L. paracasei* could be significantly greater than that in the latter, live cells^28^. Moreover, it is implied that artificial physical stress due to DNA extraction could cause additional fragmentation by the handbook manual of a typically commercial DNA extraction kit as presented in the later Methods section.

As mentioned above, concerning the extraction of chromosomal DNA from heat-killed *L. paracasei* cells in different types of NF, it is unknown whether the five copies of 16S rDNA are intact, each intact gene copy is located on the same chromosomal DNA, or whether each intact copy is located on different fragmented DNA molecules. A series of these elucidations might enable researchers to implement an absolute dPCR assay of heat-killed *L. paracasei* supplemented in NF without any standard correlative samples.

## Results

### OD_260_ measurement results for DNA purified from HK-***L. paracasei*** originally supplemented to NFs, and the relevant cells-free NFs

**Figure 1:**
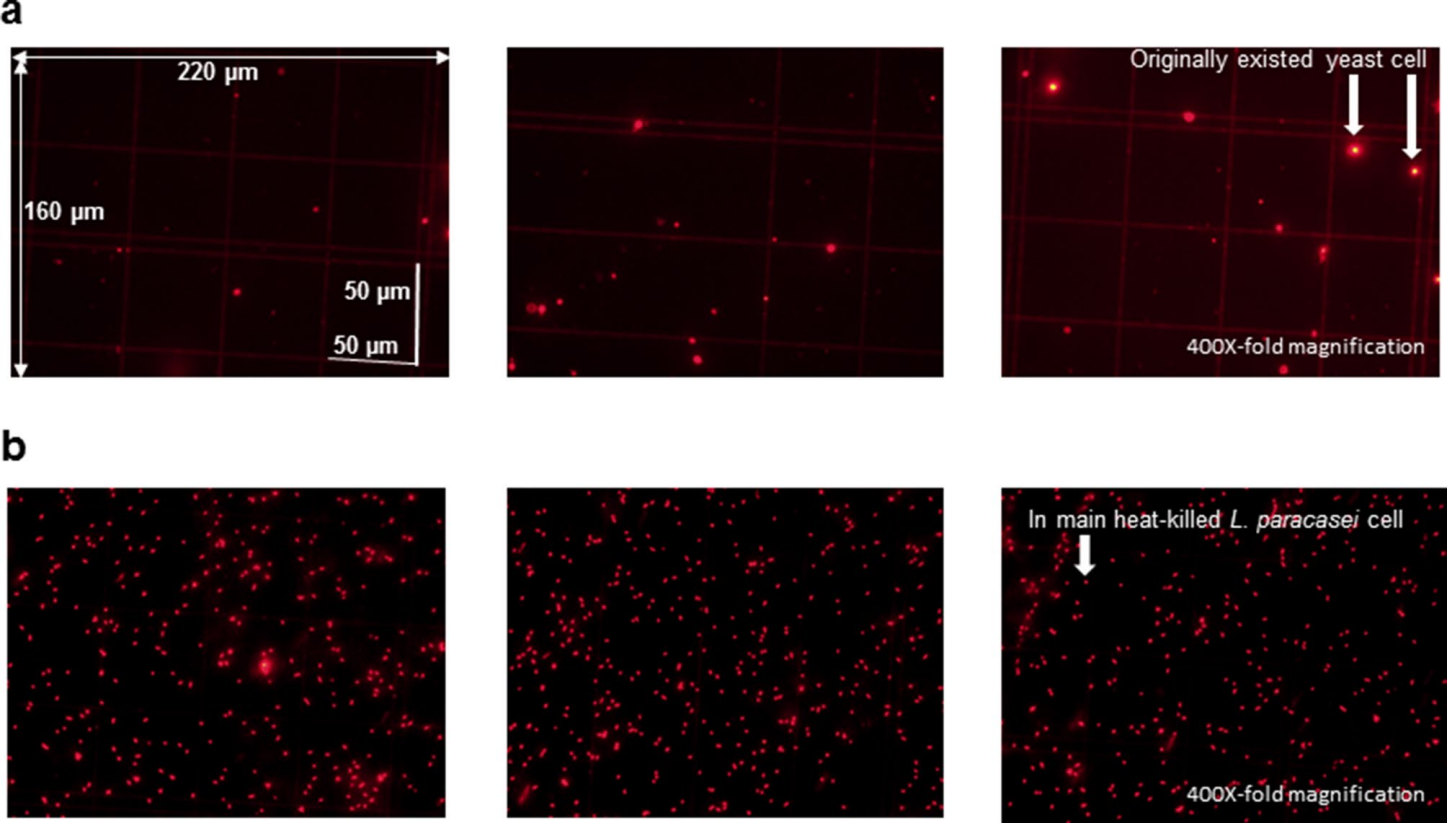
Fluorescent microscopy images with PI staining of heat-killed (HK) *L. paracasei*-free nutritional food with yoghurt flavour and relevant cell-supplemented nutritional food with yoghurt flavour. A representative three images are shown for each sample. The length and width of a microscopic field is 160 µm and 220 µm. The images were taken with × 400-fold of magnification. (**a**) HK-*L. paracasei*-free nutritional food with yogurt flavor (NFY). (**b**) HK-*L. paracasei*-supplemented NFY product (originally supplemented concentration of 2.0 × 10^8^ cells/ml).

DNA recovery rates of HK-*L. paracasei*-supplemented NFs (yoghurt flavour, strawberry flavour, milk tea flavour, or orange flavour; NFY, NFS, NFM, or NFO, respectively) using OD_260_ measurement are presented (Table 1). As implied in Fig. 1, HK-*L. paracasei*-free NFY originally contained some yeast cells (Table 1). Thus, the total DNA concentrations ranging from 2.75 to 4.45 ng/µl were obtained, and original yeast DNA concentration (from the associated free samples) was subtracted, consequently DNA concentrations of HK-*L. paracasei* alone were accurately obtained (Table 1). DNA recovery rates estimated using OD_260_ were 0.54–0.62 (NFY), 0.44–0.58 (NFS), 0.46–0.60 (NFM) and 0.26–0.40 (NFO) for different NFs, considering one cell provides 5 fg of chromosomal DNA. The results implied that a slight difference in the DNA recovery rate could occur among the same kinds of NFs. However, the variance in the recovery rate was superior to that of traditional DNA purification method using phenol/chloroform extraction (Supplementary Table S1). Regardless of the type of DNA extraction, the results implied that a traditional DNA extraction could trigger poor variance of DNA recovery.

**Table 1:** OD_260_ measurement data for chromosomal DNA that were purified from both heat-killed (HK) *L. paracasei*-supplemented nutritional foods and the relevant cell-free foods.

	Test sample	DNA extraction	Total DNA conc. (ng/µl) by OD_260_		Test sample	DNA extraction	Mainly yeast DNA conc. (ng/µl) by OD_260_
HK-*L. paracasei*-supplemented nutritional foods (2.0 × 10^8^ cells/ml)	With yogurt flavor	1st	4.45	HK-*L. paracasei*-free nutritional foods	With yogurt flavor	1st	2.95
		2nd	3.75			2nd	2.20
		3rd	3.35			3rd	2.00
	With strawberry flavor	1st	3.20		With strawberry flavor	1st	1.90
		2nd	3.30			2nd	2.20
		3rd	3.45			3rd	2.00
	With milk tea flavor	1st	3.00		With milk tea flavor	1st	1.85
		2nd	3.45			2nd	1.95
		3rd	3.20			3rd	2.05
	With orange flavor	1st	2.65		With orange flavor	1st	2.00
		2nd	2.75			2nd	1.75
		3rd	2.80			3rd	1.90

	Test sample	DNA extraction	HK-*L. paracasei* DNA conc. (ng/µl)^a^	HK-*L. paracasei* DNA recovery rate due to OD_260_^b^	HK-*L. paracasei* DNA recovery rate in triplicate		
					Mean	SD	RSD (%)
HK-*L. paracasei*-supplemented nutritional foods	With yogurt flavor	1st	1.50	0.60	0.59	0.042	7.1
		2nd	1.55	0.62			
		3rd	1.35	0.54			
	With strawberry flavor	1st	1.30	0.52	0.51	0.070	13.7
		2nd	1.10	0.44			
		3rd	1.45	0.58			
	With milk tea flavor	1st	1.15	0.46	0.51	0.081	16.0
		2nd	1.50	0.60			
		3rd	1.15	0.46			
	With orange flavor	1st	0.65	0.26	0.34	0.072	21.2
		2nd	1.00	0.40			
		3rd	0.90	0.36			

^a^True heat-killed (HK) *L. paracasei* DNA concentration was obtained by subtracting yeast DNA concentration from total DNA concentration.

^b^HK-*L. paracasei* DNA recovery rate was obtained by measuring HK-*L. paracasei* DNA concentration (ng/µl) divided by 100% recovery concentration of 2.50 ng/µl calculated using the supplemented HK-*L. paracasei* cells (2 × 10^8^ cells/ml).

### Estimated elongation rate for HK-*L. paracasei*-specific 16S rDNA and single-copy house-keeping gene *hsp60* using absolute dPCR master mix

*Lactobacillus paracasei-*specific 16S rDNA (FAM-DNA probe) and house-keeping *hsp60* gene (Hex-DNA probe) coded by the single-copy of the artificial gBlock DNA (Supplementary Fig. S1) were used. For the cDBC-included direct-dPCR master mix (direct master mix)^29–31^, 16S rDNA was mixed with the direct master mix (2000, 1,000, or 500 copies per 1 µl of the master mix). The dPCR data of 781.76 ± 69.13, 482.89 ± 18.70, or 278.90 ± 24.25 copies/µl were obtained for expected 2000, 1,000, or 500 copies per 1 µl of the master mix. Similarly, *hsp60* gene fragment was also applied to the direct master mix (2000–500 copies/µl), which gave the resulting dPCR data of 899.14 ± 72.40–311.24 ± 30.40 copies/µl (Supplementary Table S2). Thus, the mean reaction rate (%) for the direct master mix was 47.7%, almost 1.43 times higher than that of the traditional dPCR master mix without any cDBC (33.3%) (Supplementary Table S2), which implied that the direct master mix is preferable compared to the typical dPCR master mix for purified DNA but not for the DNA retained in cells (Supplementary Table S2)^29–31^. Additionally, the ratio of the 16S rDNA/*hsp*60 gene copy number was 0.9 for the direct master mix and 1.0 for the typical dPCR master mix. Their ratios implied that both master mixes could amplify both 16S rDNA and *hsp60* at almost the same elongation rate.

### Specificity, sensitivity, detection limit (LOD), and quantification limit (LOQ) for the absolute dPCR following DNA extraction using the direct master mix for *L. paracasei*

For the specific dPCR primers used to elongate the 16S rDNA of *L. paracasei* followed the previously published report by Byun et al.^32^. However, additionally, we ascertained the amplification by dPCR and the qPCR never occurred for six kinds of commercial good germ bacterial powders (*L. paracasei*-free powders), and 17 kinds of good germs and pathogens (type-strain) other than *L. paracasei* (Supplementary Table S3).

In relation to the sensitivity, accuracy, and relative specificity, following EN ISO 16140:2003 validation^33^, absolute dPCR had 100% relative sensitivity and accuracy, as well as specificity compared to the traditional qPCR with a correlation curve^33^ (Supplementary Table S4). We also estimated the detection limit (LOD: 7.10 × 10^5^ cells/ml) and quantification limit (LOQ: 2.15 × 10^6^ cells/ml) at product level (Supplementary Table S5).

### Comparison of HK-*L. paracasei* standard samples with HK-*L. paracasei*-supplemented NF in terms of DNA recovery rate (OD_260 nm_) and the 16S rDNA/*hsp60* ratio by absolute dPCR

HK-*L. paracasei* cells were exogenously added to the relevant cell-free NFs, and these were subjected to DNA isolation, and then the concentrations of chromosomal DNA were calculated (Supplementary Fig. S2) to obtain the fundamental data for the DNA recovery of HK-*L. paracasei.* Overall, the true recovery rate (42.0–58.0%) of HK-*L. paracasei* kept at 90 °C for 15 min (Supplementary Fig. S2) was close to that of HK-*L. paracasei* (Table 1) exposed to 90 °C for 15 min and an additional MDU sterilisation (140 °C for 2 s), as well as homogenisation in the final production process.

Regardless of live or HK-*L. paracasei* cells (90 °C for 15 min alone), the 16S rDNA/*hsp60* ratio (5.2–6.6; approx. 6) was slightly higher than the copy number (5) obtained by the TakaRa-Bio NGS analysis (Table 2). In contrast, the relevant ratio (5.0–7.2; maximum approx. 7) of HK-*L. paracasei* (90 °C for 15 min, additional MDU sterilisation and homogenisation) final products, was slightly higher than that of the HK-*L. paracasei* cells exposed to heat at 90 °C for 15 min (Table 2), and noticeably higher than that of the TaKaRa-NGS (Table 3).

**Table 2:** The absolute dPCR measurement results due to double colours of FAM (16S rDNA specific) and HEX (*hsp60*) DNA probes for chromosomal DNA that were extracted from live and heat-killed (HK) suspension, or HK-*L. paracasei* exogenously added to the relevant cell-free nutritional foods.

Test sample^a^	DNA extraction	16S rDNA (copies/µl) in the direct master mix	*hsp60* gene (copies/µl) in the direct master mix	Ratio (16S rDNA/*hsp60* gene)^b^	Chromosomal DNA molecules/µl in the direct master mix calculated from the absolute dPCR^c^
Live *L. paracasei*	1st	3,102.3	532.7	5.8	1,121.3
	2nd	2,800.0	466.3	6.0	978.3
	3rd	2,655.7	477.8	5.6	994.2
Material powder of M-1 powder (HK-*L. paracasei*)	1st	2,878.4	485.8	5.9	1,022.8
	2nd	2,659.3	439.5	6.1	913.9
	3rd	2,474.3	443.1	5.6	926.3
HK-*L. paracasei* exogenously added in NFY	1st	3,135.5	473.6	6.6	996.0
	2nd	2,616.4	401.2	6.5	843.9
	3rd	3,485.3	557.5	6.3	1,159.8
HK-*L. paracasei* exogenously added in NFS	1st	3,004.1	563.8	5.3	1,188.3
	2nd	2,475.3	458.7	5.4	961.0
	3rd	2,784.7	501.1	5.6	1,042.5
HK-*L. paracasei* exogenously added in NFM	1st	3,602.0	562.5	6.4	1,179.9
	2nd	3,301.1	505.3	6.5	1,064.7
	3rd	3,890.4	623.4	6.2	1,315.5
HK-*L. paracasei* exogenously added in NFO	1st	3,320.2	634.4	5.2	1,338.6
	2nd	3,104.6	572.6	5.4	1,205.3
	3rd	3,704.3	669.1	5.5	1,412.0

Purified DNA concentration used for the absolute dPCR was 0.05 ng/µl due to OD_260_, which corresponds to OD_260_-based 1.0 × 10^3^ chromosomal DNA molecules/µl in the direct master mix.

The values should be principally identical to the value, 1,000 molecules/µl of “Chromosomal DNA molecules/µl of the direct master mix due to OD_260_”.

^a^HK-*L. paracasei* cells were exogenously added in the relevant cell-free nutritional food with yogurt (NFY), strawberry (NFS), milk tea (NFM) or orange flavor (NFO). DNA purified solutions that were adjusted to 0.05 ng/µl was added to the direct master mix at a tenfold dilution.

^b^Ratio is calculated from “16S rDNA (copies/µl) in the direct master mix/*hsp60* (copies/µl) in the direct master mix”.

^c^The numerical formula is “16S rDNA (copies/µl) in the direct master mix/Ratio (16S rDNA/*hsp60* gene) × 100/mean reaction rate (%) of 47.7 with the direct master mix in Supplementary Table S2”.

**Table 3:** dPCR measurement results due to double colours of FAM (16S rDNA specific) and HEX (*hsp60*) DNA probes for chromosomal DNA that were extracted from heat-killed (HK) *L. paracasei*-supplemented nutritional food products.

Test sample^a^	DNA extraction	16S rDNA (copies/µl) in the direct master mix	*hsp60* gene (copies/µl) in the direct master mix	Ratio (16S rDNA/ *hsp60* gene)	Actually chromosomal DNA conc. with Test sample (ng/µl) due to OD_260_^b^	Chromosomal DNA in the direct master mix (molecules/µl) due to OD_260_^b^
HK-*L. paracasei-*NFY	1st	3,914.5	546.3	7.2	1.50 × 10^–1^	3.00 × 10^3^
	2nd	3,629.7	525.5	6.9	1.55 × 10^–1^	3.10 × 10^3^
	3rd	3,112.8	460.1	6.8	1.35 × 10^–1^	2.70 × 10^3^
HK-*L. paracasei*-NFS	1st	1948.9	347.9	5.6	1.30 × 10^–1^	2.60 × 10^3^
	2nd	1915.6	352.4	5.4	1.10 × 10^–1^	2.20 × 10^3^
	3rd	2,808.8	422.9	6.6	1.45 × 10^–1^	2.90 × 10^3^
HK-*L. paracsei*-NFM	1st	3,157.1	455.0	6.9	1.15 × 10^–1^	2.30 × 10^3^
	2nd	2,834.2	392.2	7.2	1.50 × 10^–1^	3.00 × 10^3^
	3rd	2,706.5	422.9	6.4	1.15 × 10^–1^	2.30 × 10^3^
HK-*L. paracasei*-NFO	1st	1,483.7	296.7	5.0	6.50 × 10^–2^	1.30 × 10^3^
	2nd	2,207.7	356.8	6.2	1.00 × 10^–1^	2.00 × 10^3^
	3rd	2,174.9	311.1	7.0	9.00 × 10^–2^	1.80 × 10^3^

Test sample^a^	DNA extraction	DNA recovery rate due to OD_260_^c^	Absolute dPCR assay (cells/ml)^d^	Absolute dPCR assay for products		
				Mean (cells/ml)	SD (cells/ml)	RSD (%)
HK-*L. paracasei-*NFY	1st	0.60	7.60 × 10^7^	7.27 × 10^7^	2.83 × 10^6^	3.9
	2nd	0.62	7.11 × 10^7^			
	3rd	0.54	7.11 × 10^7^			
HK-*L. paracasei*-NFS	1st	0.52	5.61 × 10^7^	6.17 × 10^7^	5.75 × 10^6^	9.3
	2nd	0.44	6.76 × 10^7^			
	3rd	0.58	6.15 × 10^7^			
HK-*L. paracsei*-NFM	1st	0.46	8.34 × 10^7^	7.18 × 10^7^	1.49 × 10^7^	20.8
	2nd	0.60	5.50 × 10^7^			
	3rd	0.46	7.71 × 10^7^			
HK-*L. paracasei*-NFO	1st	0.26	9.57 × 10^7^	8.09 × 10^7^	1.28 × 10^6^	15.8
	2nd	0.40	7.47 × 10^7^			
	3rd	0.36	7.24 × 10^7^			

^a^Products, nutritional foods with yogurt (NFY), strawberry (NFS), milk tea (NFM)or orange (NFO) flavour originally supplemented with HK-*L. paracasei* cells were applied to DNA extraction. In view of originally supplemented concentration of HK-*L. paracasei*, a tenfold dilution of purified DNA concentration (ng/µl) used for the absolute dPCR was 0.25 ng/µl if 100% recovery obtained, which corresponds to 5.0 × 10^3^ chromosomal DNA molecules/µl of the direct master mix.

^b^The values correspond to 1–10th of the relevant data of Table 1 owing to the tenfold dilution of purified DNA solutions. “Chromosomal DNA molecules in the direct master mix (molecules/µl) due to OD_260_” is calculated, quoting one molecule = 5 fg of chromosomal DNA for prokaryotic cell.

^c^DNA recovery rate due to OD_260_ was calculated using “Chromosomal DNA molecules in the direct master mix (molecules/µl) due to OD_260_/just upper-mentioned 5.0 × 10^3^ chromosomal DNA molecules/µl of the direct master mix”.

^d^Absolute assay data was obtained from the successive numerical formula: “16S rDNA (copies/µl) in the direct master mix/Ratio (16S rDNA/*hsp60* gene) × a dilution (tenfold) of purified DNA solution × addition of diluted DNA to the direct master mix at a tenfold dilution × purified DNA solution volume (0.2 ml)/volume for diluted test samples that were supplied for DNA extraction (1.5 ml) × original dilution factor (3) for nutritional foods × 1,000 (owing to a conversion of cells/µl to cells/ml) × 1/DNA recovery rate × 100 /Mean value of 47.7 of reaction rate (%) in Supplementary Table S2”.

### Absolute dPCR results of HK-*L. paracasei*-supplemented NFs without any standard samples following DNA extraction

Chromosomal DNA purified from HK-*L. paracasei*-supplemented NFs was simultaneously amplified by using primers for both *L. paracasei*-specific 16S rDNA, and a single-copy gene of *hsp60* (Table 3). If 100% DNA recovery was achieved, HK-*L. paracasei* 1 DNA molecule added to 1 µl of the direct master mix could become 5,000 DNA molecules. However, HK-*L. paracasei* DNA molecules that were actually added to the master mix were 2,700–3,100 (NFY), 2,200–2,900 (NFS), 2,300–3,000 (NFM), or 1,300–2,000 (NFO) DNA molecules/µl (Table 3), followed by the recovery rate actually measured using OD_260_ as presented in Table 1.

In contrast, the actual 16S rDNA measured were 3,112.8–3,914.5 (NFY), 1,915.6–2,808.8 (NFS), 2,706.5–3,157.1 (NFM), or 1,483.7–2,207.7 (NFO) copies/µl for 4 NFs products (Table 3). Likewise, obtained *hsp60* copy number using dPCR was 460.1–546.3 (NFY), 347.9–422.9 (NFS), 392.2–455.0 (NFM), or 296.7–356.8 (NFO) copies/µl for 4 NFs (Table 3). The number of chromosomal DNA per 1 µl of the master mix was obtained by dividing the 16S rDNA data (copies/µl) in Table 3 by the associated ratio (16S rDNA/*hsp60*). As presented in the below section, Methods, when considering the dilution and concentration factors of NFs sample before application to the absolute dPCR, as well as DNA recovery rate due to OD_260_ and the reaction rate (47.7% in Supplementary Table S2) due to the direct master mix, the absolute quantification data (the relevant cells/ml of four kinds of NFs) were successfully obtained without any typical correlation curves (Table 3). The absolute dPCR assayed 7.11 × 10^7^–7.60 × 10^7^ (NFY), 5.61 × 10^7^–6.76 × 10^7^ (NFS), 5.50 × 10^7^–8.3 × 10^7^ (NFM), and 7.24 × 10^7^–9.57 × 10^7^ (NFO) cells/g for 4 NFs products (Table 3).

### Validation with the direct master mix: amplification results for both 16S rDNA and *hsp60* on the artificially produced gBlock DNA using qPCR

The 16S rDNA (FAM) or *hsp60* (Hex) (10–10^7^ copies/µl) in gBlock were subjected to qPCR to obtain Ct values (Supplementary Figs. S3a,b). Both gene amplification efficiencies were almost the same as presented in the associated slopes (− 3.539 for 16S rDNA; − 3.788 for *hsp60*) and y-intercepts (38.93 for 16S rDNA; 40.32 for *hsp60* gene) (Supplementary Figs. S3a,b), and the squares (R^2^) of the correlation coefficient (R) to indicate the linearity for standard curves (0.989 for 16S rDNA; 0.999 for *hsp60*). Additionally, with respect to other single copy genes (*pheS*, *rpoB* and *tuf*), gene amplification efficiencies were indicated as the associated slopes (− 3.607 for *pheS*; − 3.521 for *rpoB*; − 4.577 for *tuf*) and y-intercepts (39.671 for *pheS*; 40.057 for *rpoB*; 46.130 for *tuf*) (Supplementary Fig. S3c–e). Although the elongation rates for *pheS* and *rpoB* were close to that of *hsp60*, the amplification rate of *tuf* was significantly lower than that of *hsp60*.

### qPCR results of HK-*L. paracasei*-supplemented NFs following DNA extraction with the use of a correlation curve

**Figure 2:**
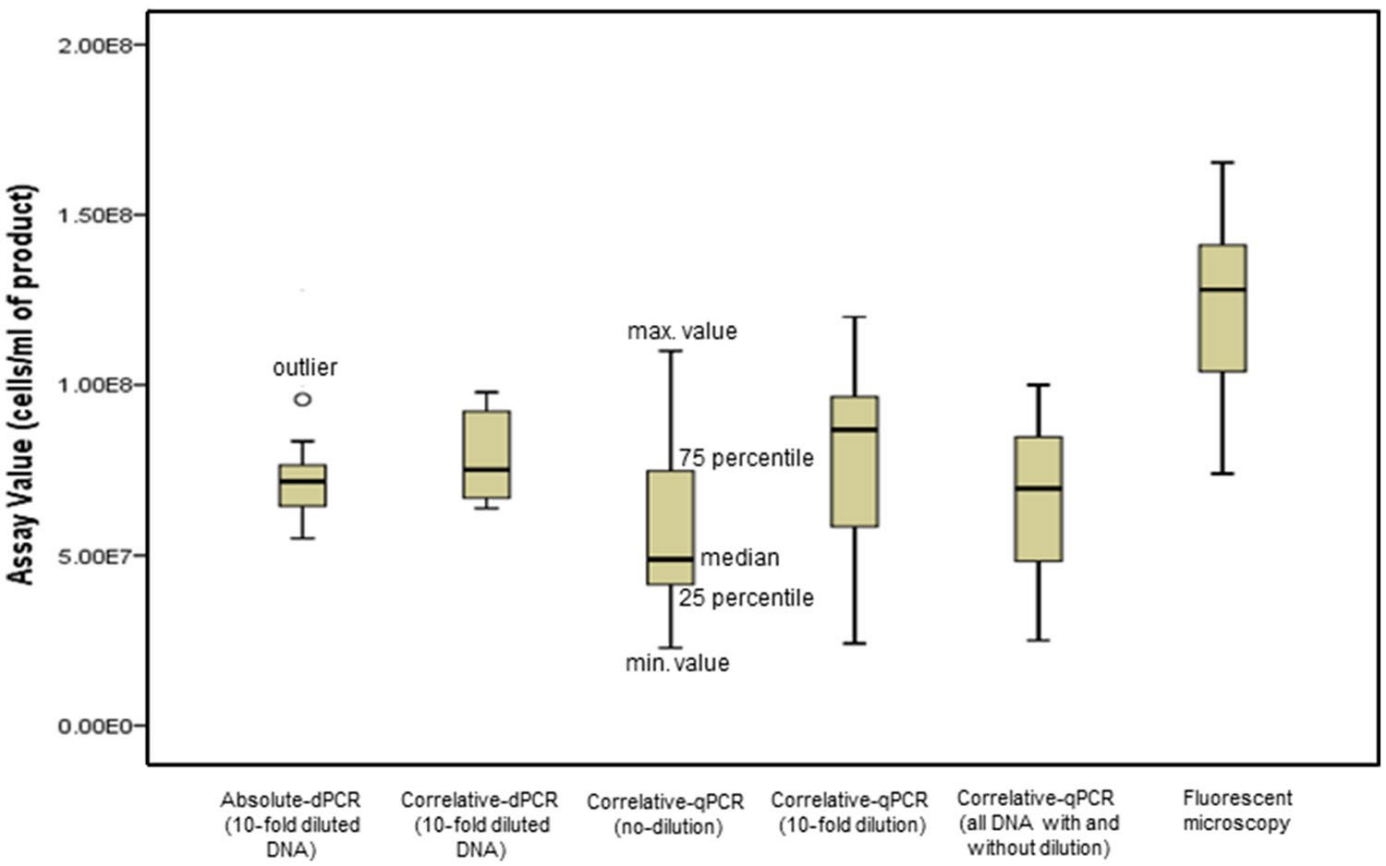
Boxplots of the absolute dPCR and traditionally used methods (the correlative dPCR, the correlative qPCR and fluorescent microscopy) of heat-killed *L. paracasei* originally supplemented in nutritional foods (with four kinds combined). Absolute-dPCR: digital PCR without any use of standard correlative samples. Correlative-dPCR: digital PCR with standard correlative samples. Correlative-qPCR: real-time PCR with standard correlative samples. Fluorescent microscopy: PI staining to specifically penetrate into heat-killed cells with standard correlative samples. With regard to dPCR and qPCR, fundamentally tenfold dilutions of purified DNA solutions were supplied for each measurement. For the correlative qPCR, the purified DNA solution itself was also supplied, and then assay data stemming from both no- and tenfold dilutions of purified DNA solution were also combined.

**Figure 3:**
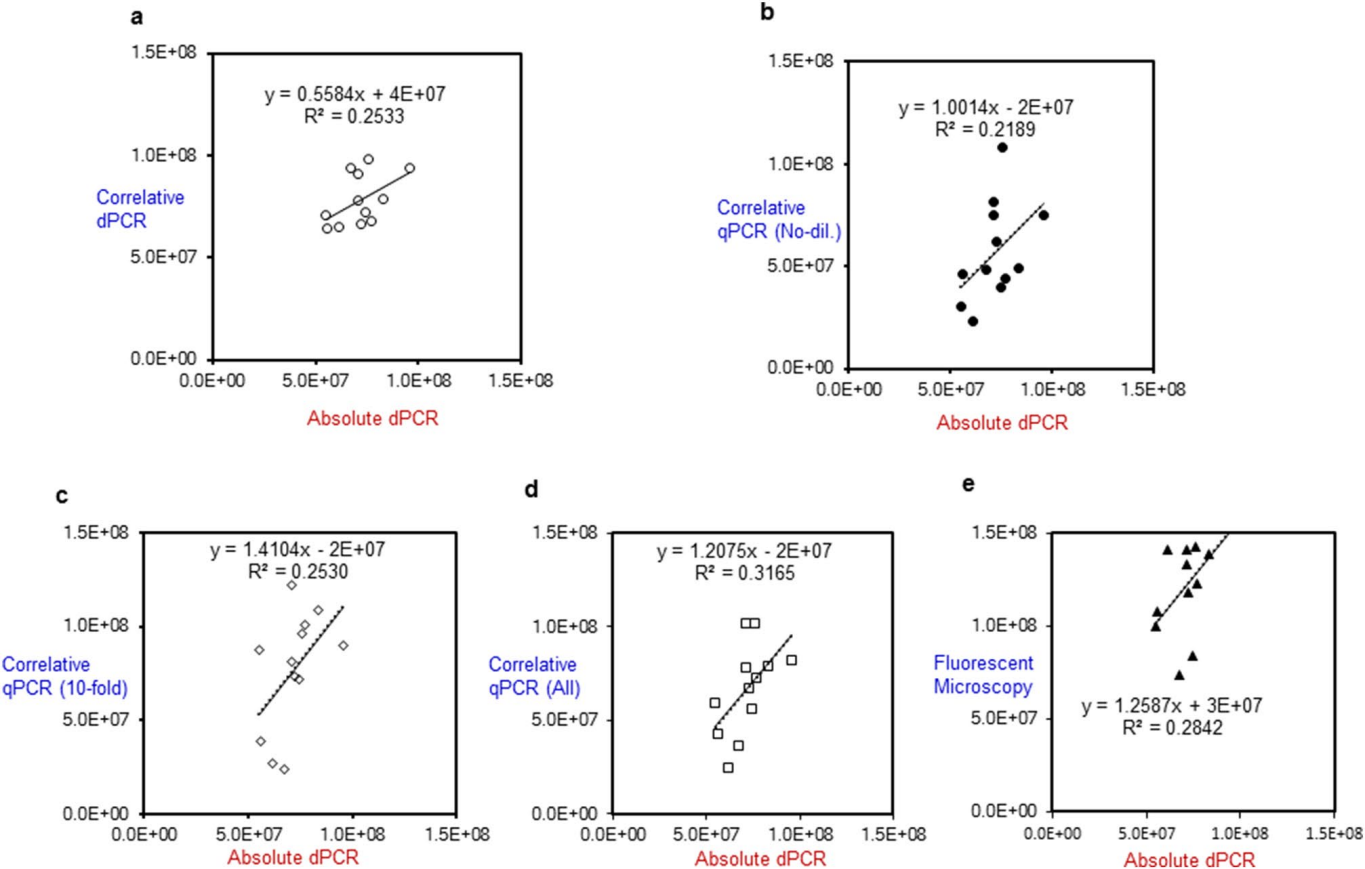
Correlative analysis between absolute dPCR and three traditional dPCR methods with a correlative standard curve, the correlative qPCR and fluorescent microscopy with a correlative standard curve for heat-killed *L. paracasei* using 4-kinds of the nutritional foods. Assay data (cells/ml) for 4-kinds of the heat-killed *L. paracasei*-supplemented nutritional food products (yogurt, strawberry, milk tea and orange flavors) were combined for each method. Absolute dPCR: digital PCR without any use of standard correlative samples. Correlative dPCR: digital PCR with standard correlative samples. Correlative qPCR: real-time PCR with standard correlative samples. Fluorescent Microscopy: PI staining to specifically penetrate into heat-killed cells with standard correlative samples. With regard to dPCR and qPCR, fundamentally tenfold dilutions of purified DNA solutions were supplied for each measurement. However, for the correlative qPCR, the purified DNA solution was also supplied, and then assay data originated from both no- and tenfold dilutions of purified DNA solution were also combined, and labeled as Correlative qPCR (All). (**a**) Absolute dPCR (X) versus Correlative dPCR (Y), (**b**) Absolute dPCR (X) versus Correlative qPCR (No-dil.), (**c**) Absolute dPCR (X) versus Correlative qPCR (tenfold) (X), (**d**) Absolute dPCR (X) versus Correlative qPCR (All) (Y), (**e**) Absolute dPCR (X) versus Fluorescent Microscopy (Y).

DNA purified from HK-*L. paracasei*-supplemented NFs were serially diluted in the relevant cell-free NF standard samples and were supplied to the traditional qPCR (Supplementary Fig. S4). The assays for *HK-L. paracasei* in NFs were performed using the aforementioned qPCR standard curves (Fig. 2; Supplementary Table S6). Similarly, concerning tenfold diluted DNA solution, the relevant assay data were obtained in 4 NFs (Fig. 2; Supplementary Table S6). With respect to the accuracy, the mean value (total of no- and tenfold diluted DNA solutions) of the assay for 4 NFs was close to that the absolute dPCR, thus correlative to the absolute dPCR. However, the interquartile range (IQR) of qPCR (all of tenfold diluted and undiluted DNA solution) in the box-plot was about three times as much as that of the absolute dPCR, which implied that both methods are in the main correlative (Fig. 3), but the precision of the absolute dPCR was superior to qPCR (Fig. 2). Incidentally, the LOD and LOQ at NFs level determined by the qPCR were approximately 2.0 × 10^2^ cells/ml and about 2.0 × 10^3^ cells/ml, respectively (Supplementary Table S7). With respect to the relevant ratios (16S rDNA/*hsp60*) determined by the qPCR compared with those in Table 2, they were 2.4–10.6, significantly lower or higher than the 16S rDNA copy number (5) by the TaKaRa-NGS, respectively. (Supplementary Table S8).

### Traditional dPCR results for HK-*L. paracasei*-supplemented NFs with the use of correlative standard curve

Standard samples, which contained a known concentration of HK-*L. paracasei* (90 °C for 15 min alone) exogenously added to the relevant cell-free NFs matrices were measured by the traditional dPCR (Supplementary Fig. S5a–d). The dPCR measurement data for the specific 16S rDNA for four kinds of HK-*L. paracasei*-supplemented NFs were 2,908.6–3,657.7 (NFY), 2,219.9–3,254.9 (NFS), 2,980.2–3,476.4 (NFM), and 2,233.6–3,161.5 (NFO) copies/µl. The assay results for the 4 NFs were calculated from the aforementioned correlative standard curves (Fig. 2 and Supplementary Table S6). With regard to the accuracy, the mean value of the total assay for four kinds of HK-*L. paracasei*-NFs was a little higher than that of the absolute dPCR, but overall, it was positively correlated with the absolute dPCR (Fig. 3). In contrast, the IQR of typical correlative dPCR box-plot was almost two times as much as that of the absolute dPCR, but was noticeably less than that of the traditional qPCR (Fig. 2). Additionally, LOD and LOQ were 1.10 × 10^5^ and 3.30 × 10^5^ cells/ml at NFs level (Supplementary Table S9).

### Fluorescent microscopy results for HK-*L. paracasei*-supplemented NFs using propidium iodide (PI) staining

Fluorescent microscopy analysis for the standard correlative samples using the same relevant cell-free NFs was performed, and good linearity was obtained (Supplementary Fig. S6a–d). Assay values for HK-*L. paracasei*-supplemented NFs using correlation curves were 1.33 × 10^8^–1.43 × 10^8^ cells/ml (− NFY), 0.74 × 10^8^–1.41 × 10^8^ cells/ml (− NFS), 1.00 × 10^8^–1.39 × 10^8^ cells/ml (− NFM), 0.84 × 10^8^–1.65 × 10^8^ cells/ml (− NFO) (Fig. 2 and Supplementary Table S6). Concerning the accuracy, the mean value for total four kinds of NFs was significantly higher than that of a series of highly specific dPCR and qPCR based on gene amplification, which implied that a non-specific reaction due to a non-specific nuclei-staining of PI could occur (Fig. 2 and Supplementary Table S6). In addition, approximate LOQ at the blending level of NFs products was calculated as 3.00 × 10^8^ cells/ml (Supplementary Table S1).

## Discussion

According to the report by the TaKaRa-Bio next-generation sequencing commissioned, a chromosomal DNA of approximately 3 × 10^6^ bp in a live *L. paracasei* MCC-1849 has five copies of 16S rDNA (approximately 1.5 × 10^3^ bp). The five copies of 16S rDNA are coded on the 109131th–110700th (1569 bp), 844227th–845796th (1569 bp), 1467616th–1469185th (1569 bp), 2513570th–2515139th (1569 bp), and 2532934th–2534503th (1569 bp) nucleotides in the genome (approximately 3 × 10^6 ^bp). Therefore, the interval lengths of adjacent 16S rDNA copies were calculated as 733,526 bp, 621,820 bp, 1,044,385 bp, and 17,795 bp.

**Figure 4:**
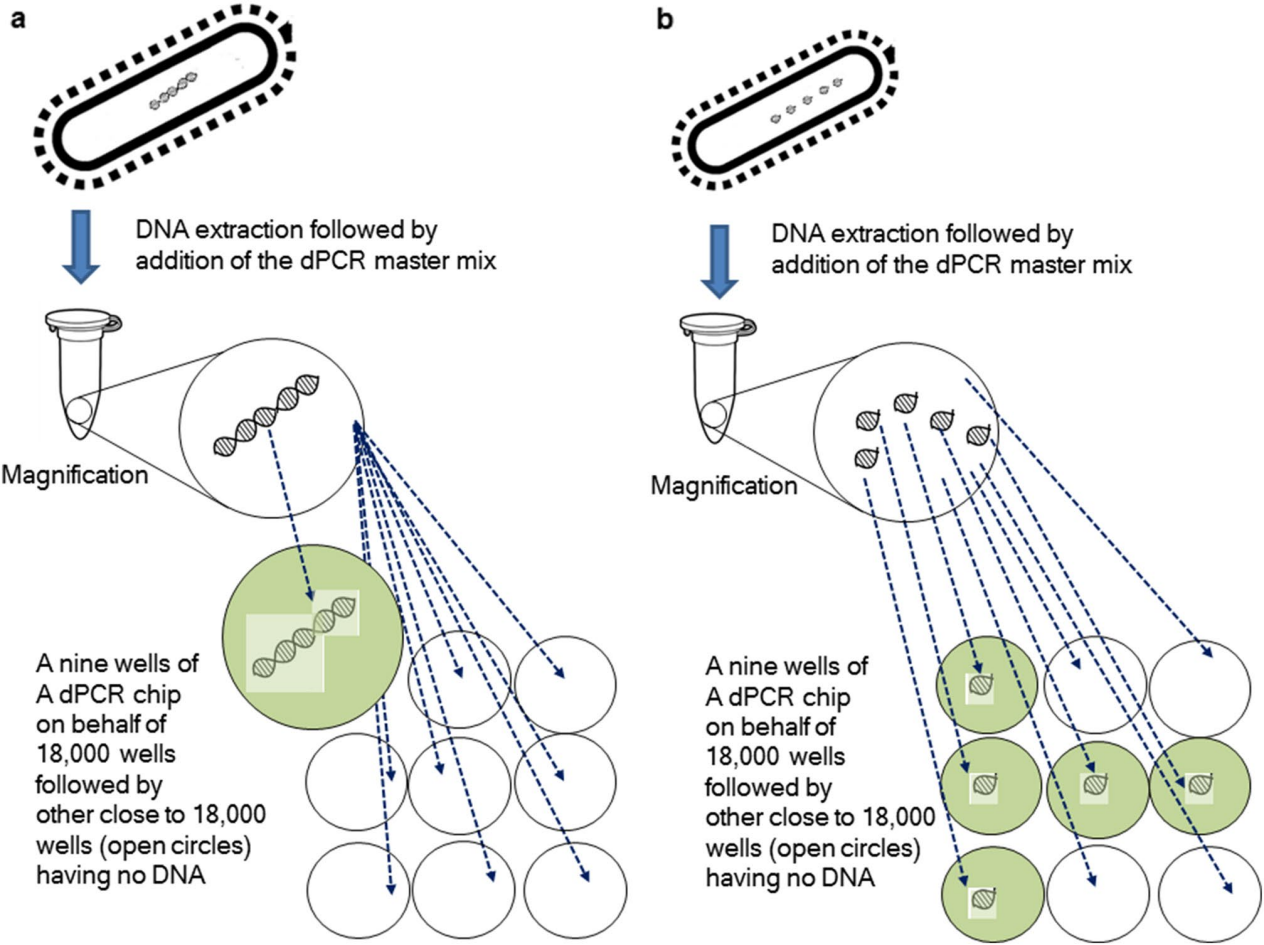
Influence of the linkage of the original five copies of 16S rDNA on the dPCR measurement data following a DNA extraction in view of the dPCR principle. 9 wells of a dPCR chip are presented representing 18,000 wells. *L. paracasei* cell originally has five copies of 16S rDNA coded on one chromosomal DNA. Upper-left image of micro-tube indicates typically used real-time PCR tube in which the dPCR master mix (direct master mix) containing template DNA (presented with open circular of ○ in its tube) was added. (**a**) Five copies of 16S rDNA coded on the same chromosomal DNA molecule. The five copies of 16S rDNA becomes dispersed to one well, which leads to a positive well, resultantly, measured as one copy. A magnified well highlighted with light-green colour contains five copies of 16S rDNA linked. A 8 wells with normal size of open circular indicates no targeted 16S rDNA copies are contained. (**b**) Five copies of 16S rDNA coded on the different relevant chromosomal DNA fragments. When the five copies of 16S rDNA are coded on the different five relevant chromosomal DNA fragments due to heat stress, the absolute dPCR determined it as five copies. Wells that contain the 16S rDNA copy at one copy are presented as closed circulars with light-green colour, and open circulars present wells that contain no 16S rDNA copies.

If relevant chromosomal DNA is isolated in the least artificially digestive manner during DNA extraction, then five copies of 16S rDNA might be located on an intact chromosomal DNA, which can be analysed by dPCR (Fig. 4a). Thus, although their five copies of 16S rDNA are in one well of the chip, the dPCR system can determine that one well is positive (resultant in one copy), using the statistical Poisson distribution if the positive rate in the wells of the dPCR chip is less than 10%^3^ (Fig. 4a). The data of one copy in the dPCR analysis is directly linked to one live *L. paracasei* cell.

In contrast, for the associated cells in NFs, an *L. paracasei* MCC-1849 cell subjected to preliminary heat at 90 °C for 15 min to prepare heat-killed *L. paracasei* cells (from frozen) is successively exposed to more heat processes during the final production process. It could cause more fragmentation (decomposition) in *L. paracasei* chromosomal DNA. If the relevant five copies of 16S rDNA are intact as a gene, but each copy is not placed on the same chromosomal DNA molecule, the five copies might be added to five different wells of the dPCR chip (the positive rate of the dPCR less than 10%^34^) (Fig. 4b). In this case, the dPCR system must consider five copies (five positive dPCR wells) per chromosomal DNA (one *L. paracasei* MCC-1849 cell), making it difficult to assay *L. paracasei* cells in NFs. As mentioned above, for an accurate assay of HK-*L. paracasei* cells in NFs, we had to elucidate whether their relevant five copies are intact or partially decomposed. If the five copies were intact, we had to successively determine how much the relevant five copies of 16S rDNA per chromosomal DNA were dispersed in dPCR wells.

To elucidate this issue, first, we had to clarify the difference between HK-*L. paracasei*-supplemented NFs and HK-*L. paracasei* exogenously added NFs, standard samples. The former was the *HK-L. paracasei* cell to sustain in final products of NFs, which had the heat exposure of 90 °C for 15 min and 140 °C for 2 s as well as homogenisation. The latter was the HK-*L. paracasei* exposed to heat (90 °C for 15 min) alone, which was exogenously added to the relevant cell-free NFs. We were motivated as we knew that the ratio of the 16S rDNA/*hsp60* was uniformly constant regardless of various thermal history for potentially differentiated foods, compared with the basic ratio of HK-*L. paracasei* that had the heat treatment (90 °C for 15 min) alone under the same NFs matrices, including the ratio of the live *L. paracasei*. However, the difference in the ratios for the former (90 °C for 15 min and 140 °C for 2 s plus homogenisation) and latter (90 °C for 15 min heat alone, including that of the live cell) instructs that its ratio must be measured per product and sample for the absolute dPCR assay (Tables 2 and 3). The ratio that practically introduces the copy number of 16S rDNA per chromosomal DNA is an important factor to accurately convert the copy number of 16S rDNA to the associated number of chromosomal DNA molecules.

In detail, when turning back to actual ratio data of 16S rDNA/*hps60*, the ratios of live *L. paracasei* were statistically almost the same as that of material powder M-1 (HK-*L. paracasei* at 90 °C for 15 min alone) as presented in Table 2. The heat at 90 °C for 15 min had a little influence on the ratio. However, the mean value of ratios for even live *L. paracasei* (5.8; approx. 6) were slightly higher than that of the 16S rDNA copy number (5) obtained by TakaRa-Bio NGS. The physical stress applied by the DNA extraction, that is, beads-beating, column centrifugation, and artificial micro-pipetting are likely to cause the slightly higher ratio for live *L. paracasei* measured by absolute dPCR. In fact, as presented in the later-section, Methods, the KURABO DNA Extraction Kit manual implies that the artificial physical stress during the DNA extraction could cause fragmentation of the DNA by nearly 1.5 × 10^4^ bp unit length. Additionally, the amplification length of the 16S rDNA was close to that of *hsp60* in this study. The *hsp60* target region may be more sensitive to artificial physical stress due to DNA extraction compared with the targeted 16S rDNA gene region. Practically, as one kind of physical stresses, the slightly higher ratios (5.0–7.2; maximum approx. 7) in Table 3 gave a hint that the *hsp60* targeted region could also be highly sensitive to additional great heating stress, MDU sterilisation (140 °C for 2 s) during the final production process compared with the targeted 16S rDNA region when compared to the 16S rDNA/*hsp60* ratio (5.2–6.6; approx. 6) in Table 2.

Importantly, the absolute assay of HK-*L. paracasei* in NFs was successfully implemented by considering the DNA recovery rate (Table 1), the amplification rate of the dPCR direct master mix (Supplementary Table S2), and the absolute dPCR based HK-*L. paracasei* chromosomal DNA molecule number per 1 µl direct master mix (Table 3).

Notably, regarding the absolute dPCR, the RSDs (%) of the DNA recovery rate for the HK-*L. paracasei*-supplemented NFs were 7.1–21.2% (Table 1), and the associated RSDs (%) of typically used DNA extraction following phenol/chloroform extraction were 17.1 to 39.0% (Supplementary Table S1). However, for our calculation method, in the absolute dPCR assay, the influence of the variance of the DNA recovery rate (Table 1 and Supplementary Table S1) was negligible. Briefly, if multiple times of DNA extractions were performed, leading to greater variance in the DNA recovery rate, then in the absolute dPCR calculations, the 16S rDNA measurement data (copies/µl) should have been the lowest in the DNA sample at the lowest DNA recovery rate.

Finally, concerning the use of *L. paracasei*-specific-16S rDNA but not a single-copy of a house-keeping gene (i.e. *hsp*60, *pheS*, *rpoB*, *tuf* or *dnaJ*) for the identification, single-copy genes for the latter are available for microbial species with whole-genome sequences^35–42^. Practically, for the identification of *Lactobacilli* using the *hsp60*, the choices are limited to restriction fragment length polymorphism following PCR (PCR–RFLP); in brief, the gene is never utilised in a typically used qPCR by not mediating RFLP^36^. When we take a closer look at the use of a single-copy gene, qPCR to target *Lactobacillus*-species-level-specific *hsp60* led to the incomplete identification of *Lactobacilli* species, and *L. paracasei* was out of scope^37^.

Additionally, the *hsp60* primers for *L. paracasei* used in this study were designed by comparing the targeted *hsp60* sequence with the whole-genome of *L. paracasei* (taxid: 1597) recorded in an NCBI Nucleotide BLAST search (https://blast.ncbi.nlm.nih.gov/Blast.cgi?PAGE_TYPE=BlastSearch). The BLAST search revealed that the *hsp60* primers in this paper could never elongate the sequence of *L. paracasei* (taxid: 1597) except for the *hsp60* region. Additionally, when considering all microbial species, the validity of their primers has not been determined. However, since NFs are targeted test samples, the non-specific amplification never occurs for the relevant cell-free NFs. Therefore, the use of *hsp60* is tolerable for the calculation of the 16S rDNA/*hsp60* ratio. Notably, our innovative dPCR method is an absolute assay without the need for a correlation curve.

## Conclusion

When PCR inhibitors existing in an NF supplemented with HK-*Lactobacilli* to improve host immune defence, DNA isolation is inevitable to circumvent under-estimation results by dPCR. For a simple, precious, and accurate routine assay of dPCR following DNA isolation, we propose the digital PCR which does not require any standard correlative samples. A multiple-copy number of 16S rDNA is frequently used to identify microbial species. We directly revealed the actual distribution degree for the 16S rDNA copies per chromosomal DNA molecule. As far as this study is concerned, the principle (the ratio: multiple-copy gene of 16S rDNA/single-copy gene of *hsp60*, *pheS* and *rpoB* other than *tuf* and *DnaJ*) is estimated to also contribute to the fulfilment of our absolute dPCR for potentially different NFs (i.e. skimmed milk powder, tofu of soybean curd, café-latte and pudding are currently under consideration, and the similar absolute dPCR evidences are about to be obtained.) with different heat treatment histories.

## Methods

### Preparation of heat-killed *L. paracasei* cells

*Lactobacillus paracasei* MCC1849 (NITE BP-01633) was heat-killed at 90 °C for 15 min following the concentration process was frozen at − 80 °C until experimentally material use.

### Preparation of test samples

After naturally thawing frozen concentrate of HK-*L. paracasei*, it was added to four kinds of the relevant-cell-free NFs (NFY to NFO) that were typically produced and sold by Morinaga Milk Industry Co., Ltd. (Tokyo, Japan). NFs were produced following the same matrices except for the flavours. For a pilot production, the HK-*L. paracasei* cells were added to the relevant cell-free NFs intermediate during the typical production process at a concentration of 2.0 × 10^8^ cells/ml followed by typical MDU sterilisation (140 °C for 2 s) and homogenisation processes, which corresponds to HK-*L. paracasei*-supplemented NFs throughout in this paper.

### Sample dilution, DNA extraction, OD_260_ measurement, calculation for the HK-*L. paracasei* DNA recovery and calculation of the relevant cells in NFs by the absolute dPCR

**Figure 5:**
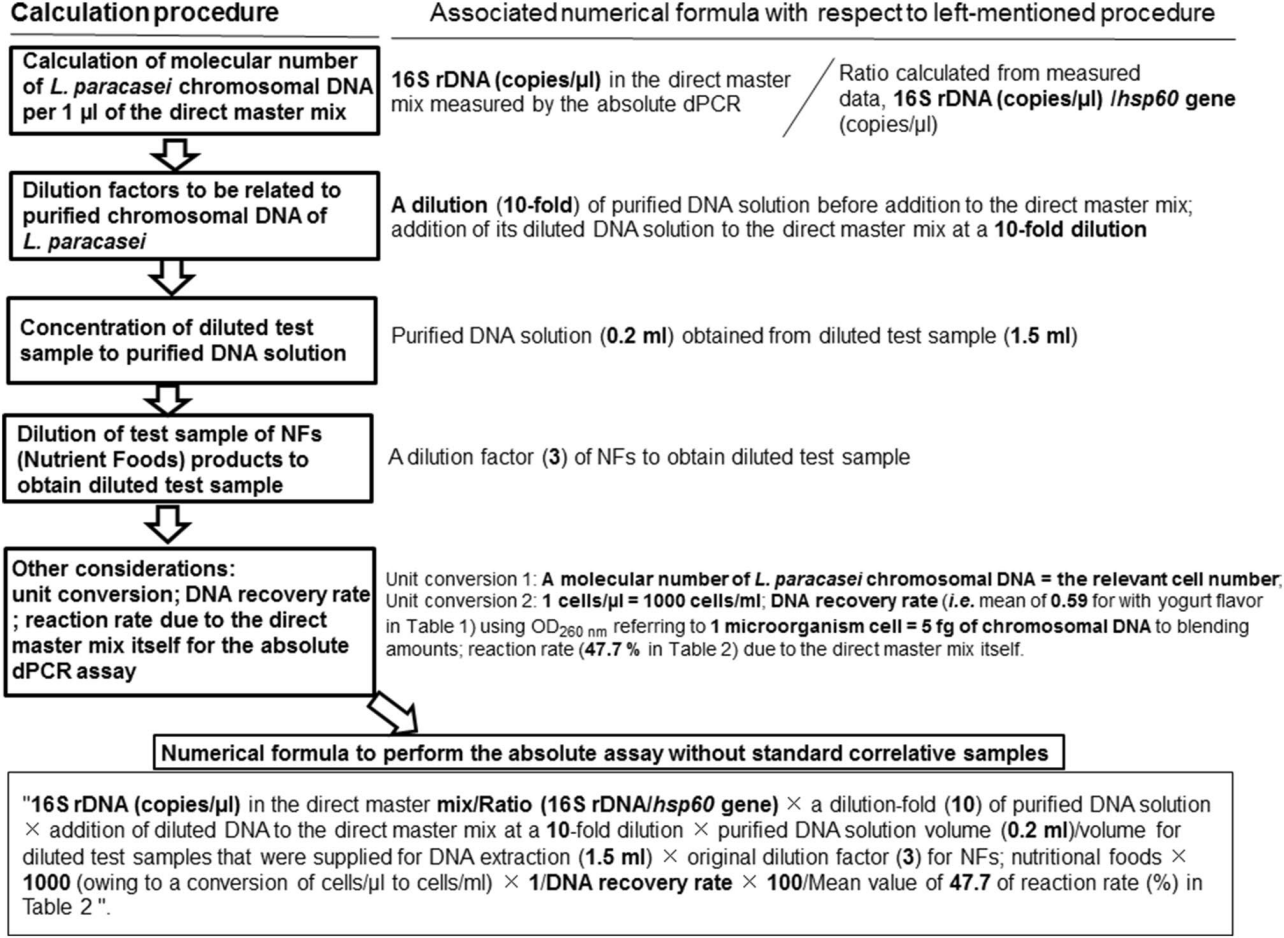
Assay principal of the absolute dPCR to target final products of NFs.

An aliquot of 10 ml of four types of HK-*L. paracasei*-free NFY to NFO, and four kinds of HK-*L. paracsei*-supplemented NFs or exogenously added NFs (products or standard samples) were diluted in 20 ml of 0.1% Tween80-PBS to obtain threefold dilution samples. An aliquot of 1.5 ml of threefold dilutions was centrifuged at 8,000×*g* for 5 min at 4 °C followed by removal of the supernatant. The zirconium beads with a diameter of 0.5 mm (450 mg) and 3.0 mm (450 mg) were added to the residual pellets in a microtube followed by vigorous vortex for 1.5 min to decompose HK-*L. paracasei* cells. Successive DNA extraction procedures following the KURABO DNA Extraction Kit (QuickGene SP kit DNA tissue; Cat. No. SP-DT) were performed, with the addition of RNase added obtain 200 µl of DNA purified solution. The OD_260_ values of HK-*L. paracasei*-supplemented NFs or exogenously added NFs (products or standards) minus that of the HK-*L. paracasei*-free NFs (mainly consisting of originally contaminated yeasts) led to the DNA concentration of HK-*L. paracasei* cells alone. HK-*L. paracasei* DNA recovery rate (%) during DNA extraction was calculated using OD_260_ values, considering the 5 fg of chromosomal DNA supplied per one HK-*L. paracasei* cell. Calculation of HK-*L. paracasei* in final products (NFs) by the absolute dPCR was performed using later-mentioned double colours of the16S rDNA (FAM)/*hsp60* (HEX) considering the reaction rate by the direct master mix itself to accurately determine a molecular number of heat-killed *L. paracasei* chromosomal DNA per 1 µl direct master mix (Fig. 5).

### Preparation of the artificially designed gBlock coding *hsp*60, *phe*S, *rpo*B, *tuf*, and *L. paracasei*-specific 16S rDNA

To evaluate how many copies of the relevant gene was actually added to the wells of the dPCR chip, the gBlock was artificially designed as described in the Supplementary Figure S1. All DNA sequences (*hsp*60, *phe*S, *rpo*B, *tuf* and *L. paracasei* 16S rDNA) were collected from the DBGET integrated database retrieval system (https://www.genome.jp/dbget/). Recorded *L. paracasei* strain p-043 DNA sequences (*hsp60*) were screened together with the whole-genome sequences of *L. paracasei* (taxid: 1597) using BLAST search (blastn suite; https://blast.ncbi.nlm.nih.gov/Blast.cgi?PAGE_TYPE=BlastSearch). Then, forward and reverse primers that could not elongate any DNA regions other than the *hsp60* region were designed. When a comparison of *hsp60* of *L. paracasei* p-043 with that of *L. paracasei* (taxid: 1597) was made, the primer regions were determined, considering varying base sequences (Supplementary Fig. S1). For specificity, the amplification of the *hsp60* using gene-specific primers of *L. paracasei* was never performed for eight kinds of the relevant cells-free NFs.

Likewise, DNA sequences of *L. paracasei* strain R-33873 (*pheS* partial), *L. paracasei* strain k-0149 (*rpoB* partial) and *L. paracasei* strain LBS3 (*tuf* partial) recorded in the DBGET were screened within the whole-genome sequences of *L. paracasei* (taxid: 1597) using BLAST. Furthermore, regions of the single-copy genes and *L. paracasei*-specific 16S rDNA was amplified using the relevant primer sets that were linked to five units of Thymidine monophosphate (TMP), as presented in Supplementary Figure S1.

Next, DNA solutions of gBlock, as presented in Supplementary Figure S1, were prepared at a concentration of 0, 5.0 × 10^3^, 1.0 × 10^4^ or 2.0 × 10^4^ molecules/µl in TE buffer, supplied by the QuickGene SP kit. The direct-dPCR master mix, comprising 10.125 µl of QuantStudio ®3D Digital PCR Master Mix v2 (Applied Biosystems, CA, USA), 0.19 µl of Platinum Taq DNA polymerase (Invitrogen by Thermo Fisher Scientific K.K., Tokyo, Japan), 1.24 µl of each of 10 µM *L. paracasei* 16S rDNA specific gene elongation forward (5′-GCACCGAGATTCAACATGG-3′; Integrated DNA Technologies, IL, USA) and reverse primers mixture (5′-GGTTCTTGGATCTATGCGGTATTAG-3′; Integrated DNA Technologies)^32^, 0.61 µl of 10 µM *L. paracasei* 16S rDNA specific TaqMan (FAM) probe (5′-/56-FAM/AACACGTGG/ZEN/GTAACCTGCCCTTAA/3IABkFQ/-3′, Integrated DNA Technologies), 1.24 µl of *hsp*-PrimeTime®STD qPCR Assay consisting of 10 µM *hsp60* forward primer (5′-GTGCTAATCCTGTTGGCATTC-3′), 10 µM *hsp60* reverse primer (5′-CCTGCGCGATTTCTTTCTTAC-3′) and 5 µM *hsp60* specific TaqMan (HEX) probe (5′-/5HEX/CTGCCGTTG/ZEN/ACGAATTGCACAAGA/3IABkFQ/-3′); Integrated DNA Technologies, 2.03 µl of cDBC components (8.3% Brij58 (Sigma-Aldrich, MO, USA), 1.9% Bovine serum albumin (Sigma-Aldrich), 10 mM Trisodium citrate dehydrate (Kanto-Kagaku, Tokyo, Japan), 30 mM MgCl_2_ (Nakarai-Tesque, Kyoto, Japan), and 100 µg/ml lysozyme from egg white (Wako, Tokyo, Japan)) as previously reported^29–31^, and 2.565 µl of distilled water.

A traditionally used dPCR master mix was prepared, substituting distilled water with cDBC components and Platinum Taq. An aliquot of 2 µl gBlock DNA solution was added to 18 µl of the cDBC-included direct-dPCR master mix, the direct master mix or the same volume of the traditional dPCR master mix was prepared, followed by successive dPCR thermal cycles by the QuantStudio™ 3D Digital PCR machine (Applied Biosystems). The thermal cycle programme for dPCR included one cycle at 96 °C for 10 min, 40 cycles at 55 °C for 30 s, 72 °C for 1 min 30 s, and 98 °C for 30 s, followed by one cycle at 72 °C for 2 min, kept at 10 °C afterwards.

### Preparation of HK-*L. paracasei* DNA standards using the relevant cell-free NFs for a correlative qPCR

The OD_260_ values of the associated HK-*L. paracasei*-free NFs were subtracted from that of relevant DNA solutions to obtain precious chromosomal DNA concentration of exogenously added HK-*L. paracasei* cells alone. The associated DNA solution (HK-*L. paracasei* alone) was serially diluted in DNA solutions that were purified from the relevant cells-free NFs to obtain 0, 0.0005, 0.005, 0.05, and 0.5 ng/µl standards for qPCR.

### The traditionally used qPCR measurements

For an applied DNA solution, DNA solutions purified from HK-*L. paracasei*-supplemented NFs products (2.0 × 10^8^ cells/ml), tenfold dilutions of their DNA solutions, those purified from relevant cell-free NFs, and standard DNA solutions (0 to 0.5 ng/µl) were selected. An aliquot of 2 µl was added to 18 µl of the direct master mix followed by the same PCR thermal cycling as mentioned before.

### The traditionally used correlative dPCR with standard correlative samples

For the standard correlative samples, HK-*L. paracasei* (frozen concentrate before use) were exogenously added to the relevant cell-free NFs at a concentration of 0, 0.5 × 10^8^, 1.0 × 10^8^, and 2.0 × 10^8^ cells/ml. Their standard correlative samples and HK-*L. paracasei*-supplemented NFs, products were diluted in 0.1% Tween80-PBS by threefolds, followed by the application of 1.5 ml of threefold diluted solution, as previously mentioned, to obtain 200 µl of purified DNA solution. The purified DNA solutions were diluted ten folds in the TE buffer, provided with the KURABO DNA Extraction Kit. Likewise, an aliquot of 2 µl was added to 18 µl of the direct master mix followed by the same PCR thermal cycling as previously mentioned.

### Fluorescent microscopy for a traditionally used photochemistry-based method

For standard correlative samples, HK-*L. paracasei* cells were artificially added to the relevant cell-free NFs at concentrations of 0, 2.5 × 10^8^, 5.0 × 10^8^, and 5.0 × 10^9^ cells/ml. An aliquot (1 ml) of the standard samples and HK-*L. paracasei*-supplemented NFs products were added to 1 ml distilled water followed by centrifugation at 2,900×*g* for 10 min at 4 °C. After the removal of the supernatant, the pellets were re-suspended in 1 ml of distilled water followed by the addition of 3 µl of twofold diluted PI (Molecular Probes Inc., USA) in the dark. After staining, the samples were kept under the safelight at approximately 25 °C for 15 min, centrifuged at 2,900×*g* for 10 min at 4 °C, followed by the removal of supernatants. Distilled water (10 ml) was added to the pellets followed by the same centrifugation. After the removal of the supernatant, the pellets were suspended in 0.5 ml of distilled water. A 2.5 µl of the tenfold diluted poly-lysine solution in distilled water was spread onto the bacteria counter (Depth of 0.020 mm; No. C9406; 1/400 & 1/16; SLGC Japan, Saitama, Japan), and it was dried. A special cover glass was overlaid and fixed to the bacteria counter, 2.5 µl of test samples with PI staining was added, and observed under a fluorescent microscope to facilitate the count of red particles alone, through the specialised filter at a 400 ×-fold magnification.

### Statistical analysis

All data analysis was performed using the IBM SPSS Statistics Ver. 24. The estimations of normality and heteroscedasticity of variances were carried out with the Shapiro–Wilk test and Levene’s test. For the assay for NFs, the differences with means of assay values obtained by each method were statistically evaluated using Bonferroni test. A P value below 0.05 was set as being statistically significant. Microsoft Office Excel 2016 was used for making linear-regression equations and lines.

## Supplementary information

Supplementary Information.

## Data Availability

All DNA sequences (*hsp*60, *phe*S, *rpo*B, *tuf* and *L. paracasei* 16S rDNA) were collected from the DBGET integrated database retrieval system (https://www.genome.jp/dbget/). Recorded *L. paracasei* strain p-043 DNA sequences (*hsp60*) were screened together with the whole-genome sequences of *L. paracasei* (taxid: 1597) using BLAST search (blastn suite; https://blast.ncbi.nlm.nih.gov/Blast.cgi?PAGE_TYPE=BlastSearch).
