# Supplementary material for: Exclusive use of digital PCR allows an absolute assay of heat-killed Lactobacilli in foods targeting multiple copies of 16S rDNA
Source: Sci Rep. 2020 Jul 29;10:12691. doi: 10.1038/s41598-020-69206-5 (PMC7391674; doi:10.1038/s41598-020-69206-5)
Supplement: Supplementary file 1 — Supplementary Information. [file 41598_2020_69206_MOESM1_ESM.pdf]

Supplementary Information (SI)

Supplementary Tables, captions, Supplementary Figures and legends

Exclusive use of digital PCR allows an absolute assay of heat-killed *Lactobacilli* in foods targeting multiple copies of 16S rDNA.

Takashi Soejima[1]*, Miyuki Tanaka[1], Koji Yamauchi[1] & Fumiaki Abe[1]

[1]Functional Food Ingredients Group, Food Ingredients & Technology Institute, R & D Division, Morinaga Milk Industry Co. Ltd., 5-1-83, Higashihara, Zama, Kanagawa 252-8583, Japan.

*Correspondence to:
Takashi Soejima
E-mail: t_soezim@morinagamilk.co.jp
TEL: +81 46(252)3045
FAX: +81 46(252)3017

Supplementary Table S1. DNA recovery rate with a typically used DNA extraction following beads-beating and phenol/chloroform extraction for 4 kinds of NFs.

		**HK-*L. paracasei* DNA recovery rate in triplicate (n = 3)**		
	Test sample	Mean	SD	RSD (%)
**HK-*L. paracasei*-supplemented product (2.0 × 10^8 cells/ml)**	Yogurt flavor	0.41	0.070	17.1
	Strawberry flavor	0.45	0.140	31.1
	Milk tea flavor	0.31	0.111	35.8
	Orange flavor	0.21	0.082	39.0

An aliquot of 10 ml of four types of HK-*L. paracasei*-free NFY to NFO, and four kinds of HK-*L. paracsei*-supplemented NFs or exogenously added NFs (products or standard samples) were diluted in 20 ml of 0.1 % Tween80-PBS to obtain 3-fold dilution samples. An aliquot of 1.5 ml of 3-fold dilutions was centrifuged at 8,000×g for 5 min at 4 °C followed by removal of the supernatant. The zirconium beads with a diameter of 0.5 mm (450 mg) and 3.0 mm (450 mg) were added to residual pellets in a microtube followed by vigorous vortex for 1.5 min to decompose HK-*L. paracasei* cells. Successive DNA extraction procedures

followed the typically used DNA extraction method using phenol/chloroform extraction followed by DNA precipitation with ethanol by Soejima *et al.* (2008) to obtain 200 µl of purified DNA solution[1].

Supplementary Table S2. dPCR measurement results for the 16S rDNA and *hsp60* coded on an artificial DNA of gBlock at one copy each with the direct master mix or typical master mix.

Test sample		dPCR master mix	Applied DNA molecules/µl of master mix	16S rDNA (copies/µl) in master mix	*hsp*60 gene (copies/µl) in master mix	Ratio (16S rDNA/*hsp*60 gene)‡	Reaction rate (%) due to master mix §	Mean of reaction rate (%)
gBlock	(2.0×10^4 molecules/µl)	Direct master mix*	2.0×10^3	781.76 ± 69.13 (n = 3)	899.14 ± 172.4 (n = 3)	0.9	39.1	47.7
	(1.0×10^4 molecules/µl)		1.0×10^3	482.89 ± 18.70 (n = 3)	550.04 ± 20.51 (n = 3)	0.9	48.3	
	(0.5×10^4 molecules/µl)		0.5×10^3	278.90 ± 44.25 (n = 3)	311.24 ± 40.40 (n = 3)	0.9	55.8	
	(2.0×10^4 molecules/µl)	Typical master mix †	2.0×10^3	385.31 ± 44.62 (n = 3)	395.11 ± 51.15 (n = 3)	1.0	19.3	17.4
	(1.0×10^4 molecules/µl)		1.0×10^3	164.63 ± 20.39 (n = 3)	164.16 ± 38.83 (n = 3)	1.0	16.5	
	(0.5×10^4 molecules/µl)		0.5×10^3	82.51 ± 14.66 (n = 3)	84.04 ± 18.87 (n = 3)	1.0	16.5	

The measurements were carried out in triplicates, and the amplification data are presented as mean ± SD (n = 3).

*The direct master mix contains Brij58, bovine serum albumin, trisodium citrate dehydrate, $MgCl_2$, and lysozyme to suppress functions of PCR inhibitors.

†Typical master mix means the commercial dPCR master mix purchased from the LifeTechnologies Inc.

‡Ratio is "16S rDNA (copies/µl) in master mix/*hsp60* (copies/µl) in master mix", in which 1.0 for the associated ratio is ideal.

§Reaction rate (%) in the master mix was calculated using "16S rDNA (copies/µl) in master mix/the associated applied DNA molecules/µl of master mix "×100.

Supplementary Table S3. Specificity for our using dPCR primers using 6 kinds of *L. paracasei*-free commercial good germ bacterial powders and 17 kinds of type-strain good germs and pathogens other than *L. paracasei*.

Tested samples	Amplification of *L. paracasei*-specific 16S rDNA
Commercial Kefir power (Mitsubishi Chemical Corporation, Tokyo, Japan)	Negative
Lactobacillus sporogenes powder (Mitsubishi Chemical Corporation, Tokyo, Japan)	Negative
BB536 powder (*Bifidobacterium longum* BB536; Morinaga Milk Industry Corp., Ltd., Tokyo, Japan)	Negative
BR-108 (heat-killed *B. longum* isolated from intestinal tract of a newborn baby, Combi Corp., Ltd., Tokyo, Japan)	Negative
EC-12 powder (heat-killed *Enterococcus faecalis*, Combi Corp., Ltd., Tokyo, Japan)	Negative
Butyrate-producing bacteria of good germ powder (Toa Pharmaceutical Co., Ltd., Tokyo, Japan)	Negative
Lactobacillus delbrueckii spp. *lactis* JCM1248T	Negative
Lactobacillus sakei ATCC15521	Negative
Leuconostoc cremoris ATCC19254	Negative
Lactobacillus acidophilus ATCC4356T	Negative
Lactobacillus plantarum ATCC14917	Negative
Lactobacillus rhamnosus ATCC53103	Negative
Enterococcus faecalis ATCC-SFA	Negative
Bifidobacterium longum ATCC15707	Negative
Escherichia. coli DH5α	Negative
Salmonella enteritidis IID604	Negative
Enterobacter sakazakii ATCC29544	Negative
Legionella pneumophila ATCC33152	Negative
Bacillus cereus ATCC14579	Negative
Listeria monocytogenes ATCC19115	Negative
Staphylococcus aureus ATCC6538P	Negative
Micrococcus luteus ATCC9341	Negative
Legionella pneumophila JLP1008	Negative

The dPCR and qPCR amplification methods followed the relevant description in the Methods section of the main text part. The measurements were carried out in three replicates.

Supplementary Table S4. Accuracy, sensitivity, and specificity of assay data of heat-killed *L. paracasei* in eight kinds of NFs products determined with the absolute dPCR relative to the typically used qPCR with correlative standard curves following EN ISO 16140:2003 validation.

Method	Sample	PA[a]	NA[a]	ND[a]	PD[a]	Sum[a]	%accuracy[b]	N^{+} [c]	%sensitivity[d]	N^{-} [e]	%specificity[f]
**Absolute dPCR**	Eight NFs	36	36	0	0	72	100.0	36	100.0	36	100.0

We assayed total of 12 samples for HK-*L. paracasei*-supplemented NFY to NFO products (triplicated sampling per food kinds and total of 4 kinds of nutritional supplementary foods), and the same number of relevant HK-*L. paracasei*-free NFY to NFO (12 samples) in the main text. Likewise, another 4 kinds of HK-*L. paracasei*-supplemented NFs (with chestnut, coffee, banana, and corn soup flavors) in six replicates per each kind, and the relevant cells-free same 4 kinds of products in six replicates were assayed to totally obtain 72 data.

[a] PA, number of samples showing positive agreement; PD, positive deviation; NA, number of samples showing negative agreement; ND, negative deviation; Sum, total numbers of samples.

[b] % accuracy data were calculated as follows: 100 _ (PA + NA)/Sum.

[c] N^{+} data were calculated as follows: PA + ND.

[d] % sensitivity data were calculated as follows: 100 × PA/N^{+}.

[e] N^{-} data were calculated as follows: NA + PD.

[f] % specificity data were calculated as follows: 100 × NA/N^{-}.

Supplementary Table S5. The LOD and LOQ at NFs products by the absolute dPCR

LOD (cells/ml) at NFs products*	LOQ (cells/ml) at NFs products†
**7.10×10^{5}**	2.15×10^{6}

The LOD and LOQ (cells/ml) for HK-*L. paracasei*-supplemented NFs (NFY to NFO) products by the absolute dPCR was estimated using 10-fold dilutions of the purified DNA solution of HK-*L. paracasei* NFs products. Concretely, twelve of the raw dPCR measurement data (copies/µl of the master mix) to elongate HK-*L. paracasei* specific 16S rDNA and *hsp60* gene for 4 kinds of the relevant cell-free NFY to NFO in triplicate were 3.66 ± 3.348 copies/µl (n = 12; mean ± SD) for the former and 3.19 ± 2.885 copies/µl (n = 12) for the latter, which stemmed from the residual fluorescence emitted by the natural restriction of FAM- or Hex-modified DNA probes.

*The LOD (cells/ml) = (2 × 10^8) / (10 (fold) × 296.7 (the lowest *hsp*60 measurement value in Table 3) / 10.53 (LOD = 3.19 × 3.3; copies/μl)) was calculated to 7.10 × 10^5 cells/ml at NFs products.

†Following the calculation method of the LOD, the LOQ was also calculated to 2.15 × 10^6 cells/ml at NFs products with the exception of LOQ = 3.19 × 10; copies/μl but not LOD = 3.19 × 3.3.

Supplementary Table S6. Assay results for heat-killed (HK) *L. paracasei* originally supplemented in NFs products by the innovative absolute dPCR and typically used methods: the correlative dPCR, the correlative qPCR and fluorescent microscopy with standard correlative samples.

Test sample	DNA extraction	Absolute dPCR (cells/ml)	Correlative analysis for dPCR* (cells/ml)	Correlative analysis for qPCR † (No-diluted DNA) (cells/ml)	Correlative analysis for qPCR † (10-fold diluted DNA) (cells/ml)	Correlative analysis for qPCR (average) ‡ (cells/ml)	Fluorescent Microscopy § (cells/ml)
NFY: Nutritional food with yogurt flavor	1st	7.60 × 10^7	9.79 × 10^7	1.08 × 10^8	9.61 × 10^7	1.02 × 10^8	1.43 × 10^8
	2nd	7.11 × 10^7	9.07 × 10^7	8.11 × 10^7	1.22 × 10^8	1.02 × 10^8	1.41 × 10^8
	3rd	7.11 × 10^7	7.78 × 10^7	7.47 × 10^7	8.14 × 10^7	7.80 × 10^7	1.33 × 10^8
NFS: Nutritional food with strawberry flavor	1st	5.61 × 10^7	6.39 × 10^7	4.60 × 10^7	3.90 × 10^7	4.25 × 10^7	1.08 × 10^8
	2nd	6.76 × 10^7	9.36 × 10^7	4.85 × 10^7	2.38 × 10^7	3.62 × 10^7	7.40 × 10^7
	3rd	6.15 × 10^7	6.50 × 10^7	2.28 × 10^7	2.68 × 10^7	2.48 × 10^7	1.41 × 10^8
NFM: Nutritional food with milk tea flavor	1st	8.34 × 10^7	7.89 × 10^7	4.91 × 10^7	1.09 × 10^8	7.92 × 10^7	1.39 × 10^8
	2nd	5.50 × 10^7	7.09 × 10^7	3.03 × 10^7	8.75 × 10^7	5.89 × 10^7	1.00 × 10^8
	3rd	7.71 × 10^7	6.77 × 10^7	4.36 × 10^7	1.01 × 10^8	7.25 × 10^7	1.23 × 10^8
NFO: Nutritional food with orange flavor	1st	9.57 × 10^7	9.36 × 10^7	7.51 × 10^7	8.98 × 10^7	8.24 × 10^7	1.65 × 10^8
	2nd	7.47 × 10^7	7.25 × 10^7	3.93 × 10^7	7.20 × 10^7	5.57 × 10^7	8.38 × 10^7
	3rd	7.24 × 10^7	6.61 × 10^7	6.18 × 10^7	7.32 × 10^7	6.75 × 10^7	1.18 × 10^8

*Correlative dPCR: Typically used dPCR with the standard correlative samples.

†Correlative qPCR assay: Typically used qPCR with the standard correlative samples. Purified DNA solution and 10-fold dilutions were supplied for the direct master mix.

‡The assay values are presented by combining mean assay values of no- and 10-fold dilution of purified DNA solutions.

§Fluorescent microscopy: Typically used fluorescent microscopic testing following PI staining with the standard correlative samples.

Supplementary Table S7. LOD and LOQ for the qPCR with the standard correlative samples.

LOD (cells/ml) at NFs products*	LOQ (cells/ml) at NFs products†
2.00×10^2	2.00×10^3

*With respect to 10^5-fold dilution DNA purified solutions obtained from HK-*L. paracasei*-supplemented NFs (NFY to –NFO), their amplifications in triplicates were successful to obtain Ct mean values of 33.11 (NFY), 35.79 (NFS), 38.60 (NFM), and 38.18 (NFO). At a range from no-dilution to 10^5-fold dilution with purified DNA solution, the degree of Ct increases was positively proportional to the increased logarithmic dilution factors. With regard to 10^6-fold dilutions, positive amplifications were done for 6 out of total 12 samples of NFs products.

†The LOQ was calculated to about 2.00×10^3 cells/ml at total of 4 kinds of HK-*L. paracasei*-supplemented NFs products[26].

Supplementary Table S8. The ratio (16S rDNA/*hsp60*) data calculated from the typically used qPCR measurement data using standard NFs samples.

Test sample*	Applied DNA molecules/µl of the DNA purified soln. due to $OD_{260\ nm}$	Mean for Ct (16S rDNA)	Mean for 16S rDNA copies/µl of the tested DNA soln.†	Mean for Ct (*hsp60*)	Mean for *hsp60* gene copies/µl of the tested DNA soln.‡	Mean for Ratio (16S rDNA /*hsp60* gene)§
HK-*L. paracasei* added	1.00×10^5	18.26	7.06×10^5	21.44	9.62×10^4	7.3
in the relevant cell-free	1.00×10^4	20.97	1.21×10^5	24.95	1.14×10^4	10.6
nutritional food with	1.00×10^3	23.63	2.10×10^4	27.36	2.60×10^3	8.1
yogurt flavor	1.00×10^2	26.38	4.00×10^3	29.98	5.00×10^2	8.0
HK-*L. paracasei* added	1.00×10^5	17.19	1.42×10^6	19.47	3.19×10^5	4.5
in the relevant cell-free	1.00×10^4	20.19	2.01×10^5	22.46	5.18×10^4	3.9
nutritional food with	1.00×10^3	23.12	2.99×10^4	25.92	6.32×10^3	4.7
strawberry flavor	1.00×10^2	26.19	4.10×10^3	29.52	7.10×10^2	5.8
HK-*L. paracasei* added	1.00×10^5	16.87	1.74×10^6	18.93	4.42×10^5	3.9
in the relevant cell-free	1.00×10^4	20.67	1.47×10^5	22.19	6.10×10^4	2.4
nutritional food with milk	1.00×10^3	24.95	9.09×10^3	27.56	2.33×10^3	3.9
tea flavor	1.00×10^2	29.47	4.80×10^2	33.15	7.8×10^2	6.2
HK-*L. paracasei* added	1.00×10^5	16.74	1.90×10^6	18.98	4.29×10^5	4.4
in the relevant cell-free	1.00×10^4	20.43	1.72×10^5	23.20	3.30×10^4	5.2
nutritional food with	1.00×10^3	24.16	1.52×10^4	27.52	2.39×10^3	6.4
orange flavor	1.00×10^2	28.34	1.00×10^3	32.08	1.50×10^2	6.7

DNA purifications were done in triplicates.

*Standard samples newly added with heat-killed *L. paracasei* of 2.0 $\times10^8$ cells/ml was diluted at a 3-fold, and an aliquot of 1.5 ml was supplied for the DNA extraction. Serially diluted DNA solutions were applied for the qPCR measurement.

† "16S rDNA copies/µl of the tested DNA soln." was calculated, using a correlative standard curve for the 16S rDNA coded on the gBlock of the artificially designed DNA at one copy as presented in Supplementary Fig. S3a.

‡ "*hsp60* gene copies/µl of the tested DNA soln." was estimated, utilizing a correlative standard curve for the hsp60 gene that was coded on the gBlock at one copy as indicated in Supplementary Fig. S3b

§"Ratio (16S rDNA/*hsp60* gene)" is "16S rDNA copies/µl of the tested DNA soln"/"*hsp60* gene copies/µl of the tested DNA soln."

Supplementary Table S9. LOD and LOQ for the dPCR method with the standard correlative samples.

LOD (cells/ml) at NFs products*	LOQ (cells/ml) at NFs products†
1.10×10^5	3.33×10^5

The LOD and LOQ for the dPCR with the specific 16S rDNA were calculated to 10.53 and 31.90 copies/µl of the direct master mix, using the relevant cell-free NFs. Supplementary Figure S5b indicated the lowest dPCR measurement value with 16S rDNA elongation for HK-*L. paracasei*-supplemented NFS (originally blending of 2.0×10^8 cells/ml HK-*L. paracasei* in NFS product; 10-fold dilution of 1st-purified DNA solution applied for the dPCR) was 1915.6 copies/µl of the direct master mix.

*The LOD (cells/ml) = $(2.0 \times 10^8)/(10$ (fold) × 1915.6 (the lowest dPCR value in 4 NFs)/10.53 (LOD copies/µl))= 1.10×10^5 cells/ml at 4 kinds of NFs products.

†The LOQ (cells/ml) = $(2.0 \times 10^8)/(10$ (fold) × 1915.6 (the lowest dPCR value)/ 31.90 (copies/µl)) = 3.33×10^5 cells/ml at four NFs products.

Table S10. LOD and LOQ for the fluorescent microscopy with the standard correlative samples.

LOD (cells/ml) at NFs products*	LOQ (cells/ml) at NFs products†
1.42×10^8	3.00×10^8

According to the manufacturer instruction of BACTERIA COUNTER (Depth of 0.020 mm; No. C9406; 1/400 & 1/16; SLGC Japan, Saitama, Japan), it is inevitable for a precious and accurate counting that 165 µm × 220 µm of the fluorescent microscopic field has approximately 70 to 140 microbial cells. As the depth for the BACTERIA COUNTER was 1/50 mm, targeted bacterial cells in the test solution that was applied on its bacterial counter required 70 to 140 × 1.4×10^6 cells/ml = approximately 1 to 2 × 10^8 cells/ml for a precious and accurate quantification. According to later-mentioned Supplementary Fig. S6a, b, c and d, the fluorescent microscopic counts were 46.4 ± 6.64 (mean ± SD, n = 12) for HK-*L. paracasei*-supplemented NFS (NFY to NFO) products.

*Considering the later-mentioned LOQ (3.0×10^8 cells/ml), as presented in the main text part, the mean value of heat-killed *L. paracasei*-free 4 kinds of NFs is 0 cells per microscopic field. The LOD following the normal distribution to indicate the population with the relevant cell-free NFs is estimated as mean (0) + 10 × SD of its normal distribution = 70 cells per microscopic field. The LOD at NFs products was calculated to 1.42×10^8 (cells/ml) of originally supplementation concentration to products × 33 (required the lowest microbial cells per microscopic field)/46.4 (actually obtained mean counts for products) = 3.00×10^8 cells/ml at total of 4 kinds of NFs products.

†Approximate LOQ at NFs products was calculated to 2.0×10^8 (cells/ml) of originally supplementation concentration to products × 70 (required the lowest microbial cells per microscopic field)/46.4 (actually obtained mean counts for products) = 3.00×10^8 cells/ml at total of 4 kinds of NFs products.

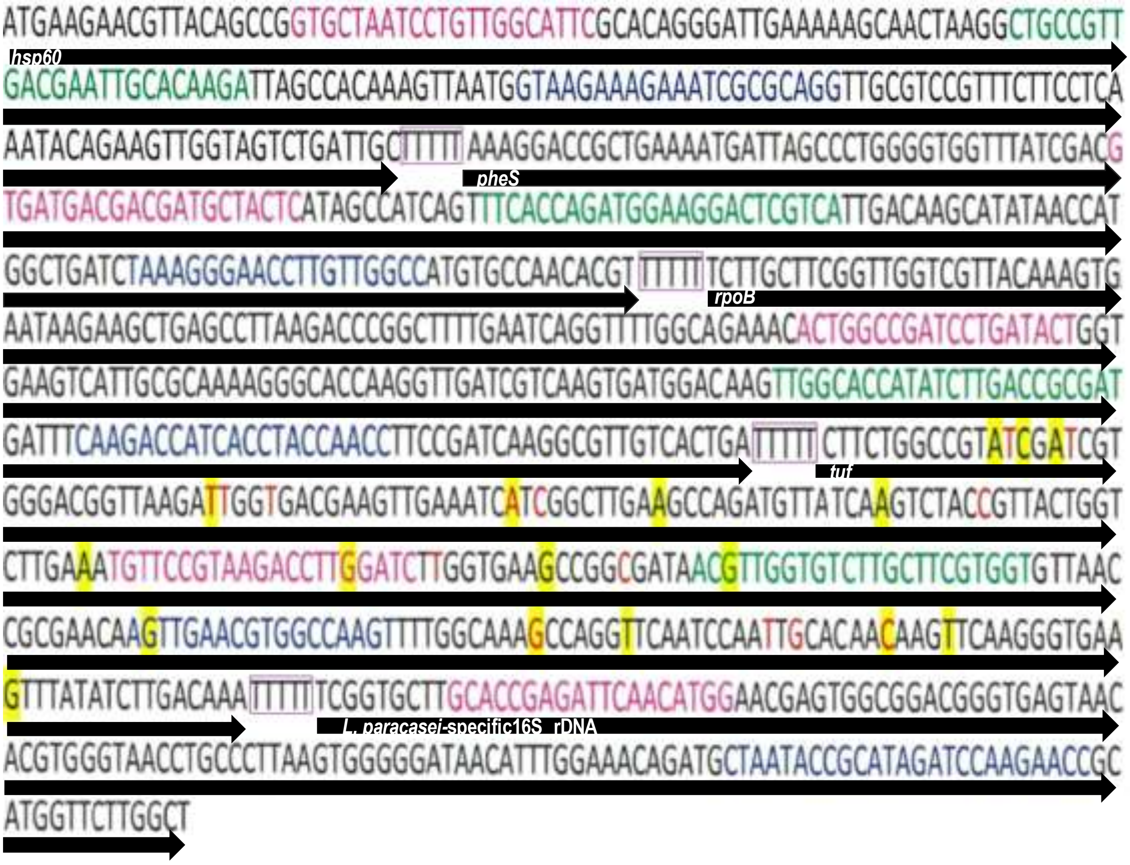

Supplementary Fig. S1. DNA sequences of the artificially designed gBlock on which not only the *L. paracasei*-specifc 16S rDNA but also *hsp60*, *pheS*, *rpoB* and *tuf* in view of Bioinfomatics using BLAST Search coded.

hsp60: gene sequences for *L. paracasei* strain p-043; *pheS*: partial sequences for *L. paracasei* strain R-33873; *rpoB*: partial sequences for *L. paracasei* strain k-0149; *tuf*: partial sequences for *L. paracasei* strain LBS3; *L. paracasei*-specific 16S rDNA: gene sequences according to the report by Byun, R. *et al.* (2004); gBlock: aforementioned four genes were linked by mediating five nucleotides with thymine alone (TTTTT). All DNA sequences with *L. paracasei* were collected from the DBGET integrated database retrieval system (https://www.genome.jp/dbget/).

DNA sequences (*hsp60*) presented with *L. paracasei* strain p-043 were screened to whole-genome sequences with *L. paracasei* (taxid: 1597) by BLAST Search (blastn suite; https://blast.ncbi.nlm.nih.gov/Blast.cgi?PAGE_TYPE=BlastSearch), and then forward and reverse primers set could not elongate any DNA sequence regions other than *hsp60* gene region in view of

bioinformatics. After a comparison of *hsp60* of *L. paracasei* p-043 with that of *L. paracasei* (taxid: 1597) was done, a region of conservative base sequences was coded on the gBlock.

pheS partial presented with *L. paracasei* strain R-33873 were screened against *L. paracasei* (taxid: 1597), and relevant primers could not elongate DNA sequences other than *pheS* for bioinformatics. After comparing the *pheS* of *L. paracasei* strainR-33873 with that of other *L. paracasei* (taxid: 1597), a conservative region was coded on the gBlock.

Likewise, *rpoB* partial of *L. paracasei* strain k-0149 were screened to *L. paracasei* (taxid: 1597), and then relevant primers could not elongate DNA sequences other than *rpoB*. Following a comparison of *rpoB* of *L. paracasei* k-0149 with that of *L. paracasei* (taxid: 1597) carried out, there were no varied base sequences.

Also, *tuf* partial of *L. paracasei* strain LBS3 were screened against *L. paracasei* (taxid: 1597), and associated primers could not elongate DNA sequences other than *tuf* for bioinformatics. Highlighted bases with red color corresponded to changeable parts, comparing the *pheS* of *L. paracasei* strainR-33873 with strain NCC2461, NCC989, X24 and W29B including *L. paracasei* (taxid: 1597). Similarly, base sequences highlighted with yellow-color were identical to varied parts when comparing the *tuf* partial of *L. paracasei* strain LBS3 with that of *L. paracasei* X3B.

Incidentally, for specificity on an experimental aspect, the amplification of aforementioned five genes specific with *L. paracasei* were also never done for 8 kinds of the relevant cells-free nutritional foods with yogurt flavor, strawberry flavor, milk tea flavor, or orange flavor including additionally another 4 flavors used in this study.

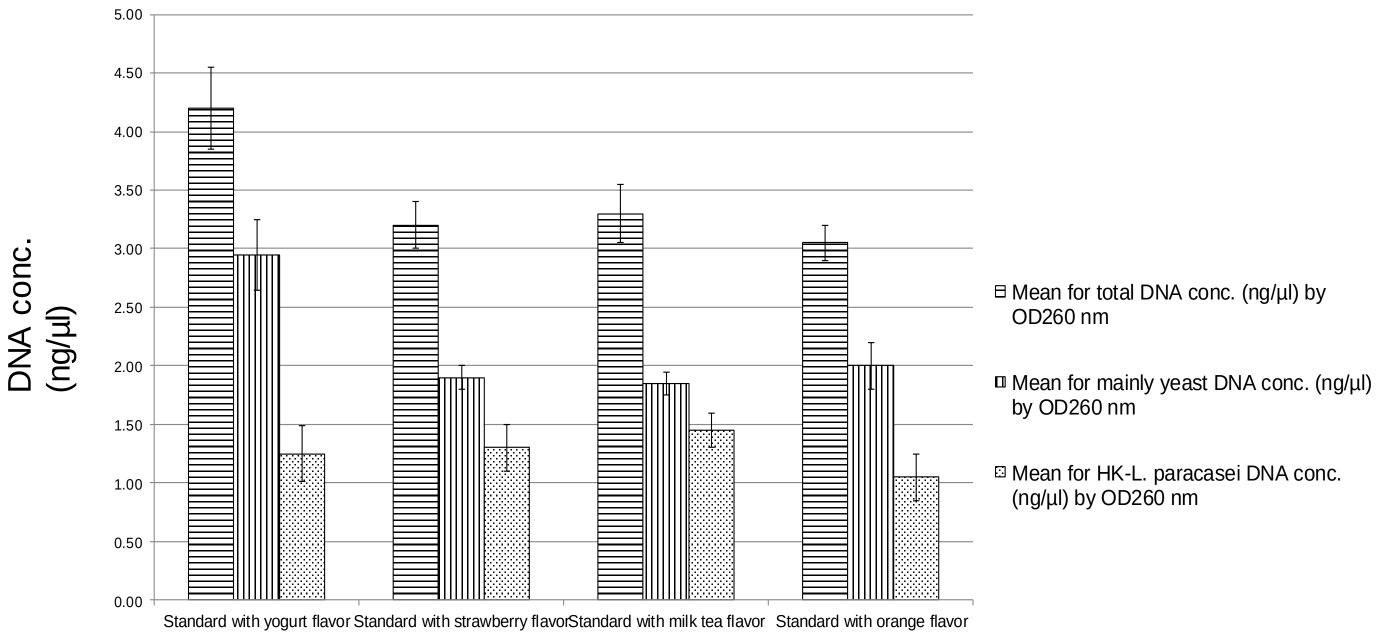

**Mean for HK-*L. paracasei* chromosomal DNA recovery rate (%) to the blending amount with standards**	**50.0 %**	**52.0 %**	**58.0 %**	**42.0 %**

Supplementary Fig. S2. OD_{260} measurement results for chromosomal DNA extracted from standard samples, to which known concentrations (2.0×10^8 cells/ml) of heat-killed (HK) *L. paracasei* (frozen concentrate) were exogenously added, and from relevant-cell-free nutritional foods (in main yeast cells).

DNA extractions were carried out in three replicates (n = 3). Each DNA concentration is presented as mean (bar) ± SD (error bar) in triplicates. A 100 % recovery for chromosomal DNA of HK-*L. paracasei* corresponds to 2.5 ng/µl. A bar with horizontal stripes is the mean for total DNA concentration (ng/µl) by OD_{260}. A bar with vertical stripes is the mean for mainly yeast DNA concentration (ng/µl) by OD_{260}. A bar with black spots is the mean for HK-*L. paracasei* DNA concentration (ng/µl) by OD_{260}.

Standard with yoghurt flavour: HK-*L. paracase*i (2.0×10^8 cells/ml) exogenously added in the relevant cell-free-nutritional foods with yoghurt flavour.

Standard with strawberry flavour: HK-*L. paracase*i (2.0×10^8 cells/ml) exogenously added in the relevant cell-free-nutritional foods with strawberry flavour.

Standard with milk tea flavour: HK-*L. paracase*i (2.0×10^8 cells/ml) exogenously added in the relevant cell-free-nutritional foods with milk tea flavour.

Standard with orange flavour: HK-*L. paracase*i (2.0×10^8 cells/ml) exogenously added in the relevant cell-free-nutritional foods with orange flavour.

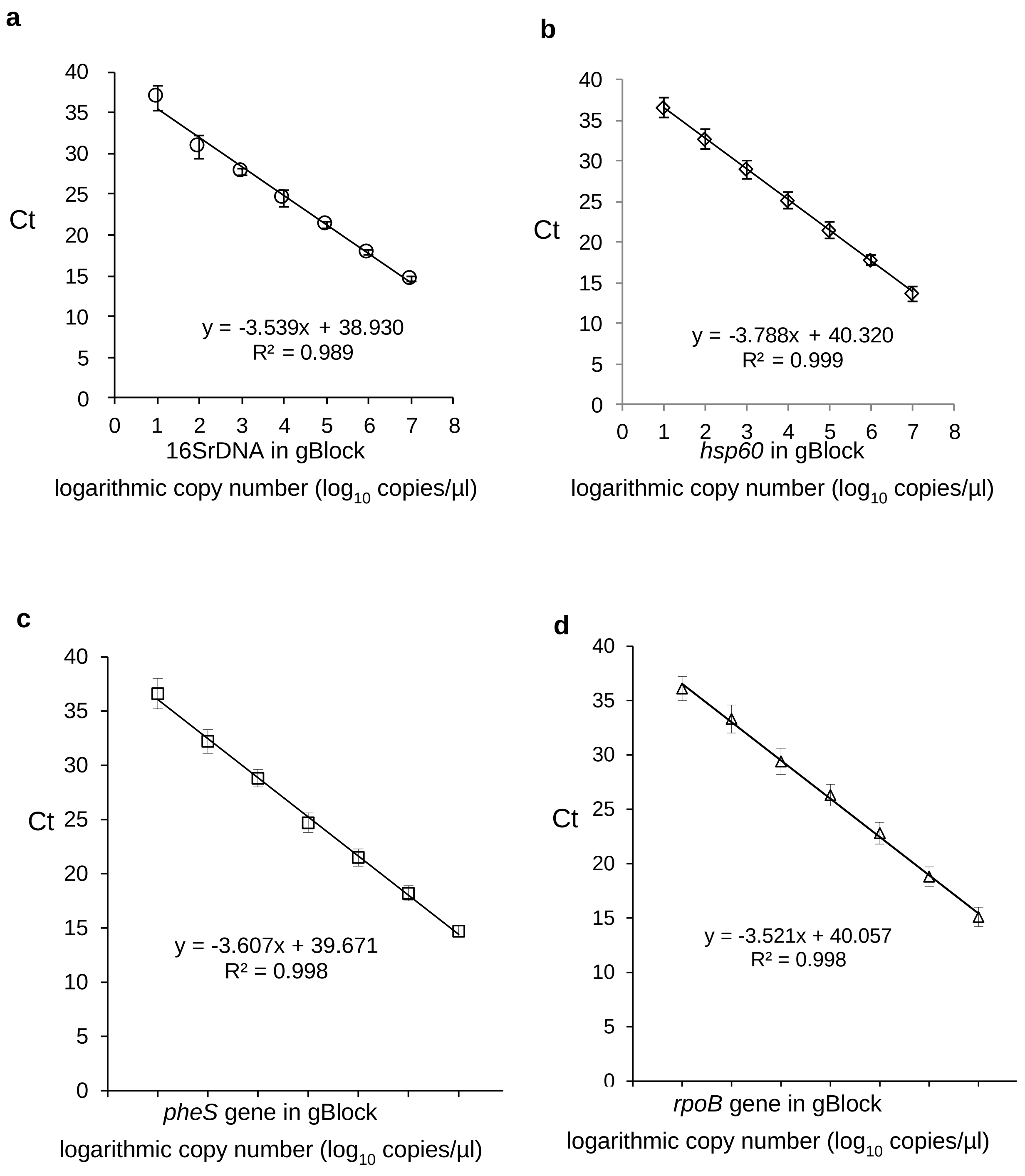
a
Ct
y = -3.539x + 38.930
R² = 0.989
16SrDNA in gBlock
logarithmic copy number (log10 copies/μl)
b
Ct
y = -3.788x + 40.320
R² = 0.999
hsp60 in gBlock
logarithmic copy number (log10 copies/μl)
c
Ct
y = -3.607x + 39.671
R² = 0.998
pheS gene in gBlock
logarithmic copy number (log10 copies/μl)
d
Ct
y = -3.521x + 40.057
R² = 0.998
rpoB gene in gBlock
logarithmic copy number (log10 copies/μl)

e

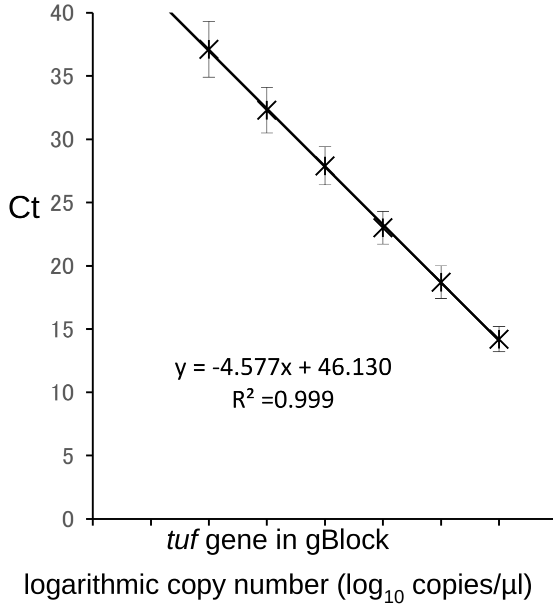

Supplementary Fig. S3. Standard curve of the 16S rDNA, *hsp60*, *pheS, rpoB and tuf* (HEX) coded on the gBlock using the qPCR.

The logarithmic copy numbers with each gene coded on the gBlock for standard samples are presented on the X-axis, and then the correlative Ct values due to the qPCR are plotted on the Y-axis. The measurements for standard samples were done in three replicates (n = 3), and then the relevant Ct values are presented as mean (○, ◇, □, △ and ×) ± SD (error bar) in triplicates.

(**a**) Standard curve with 16SrDNA gene in gBlock.

(**b**) Standard curve with *hsp60* gene in gBlock.

(**c**) Standard curve with *pheS* gene in gBlock.

(**d**) Standard curve with *rpoB* gene in gBlock.

(**e**) Standard curve with *tuf* gene in gBlock.

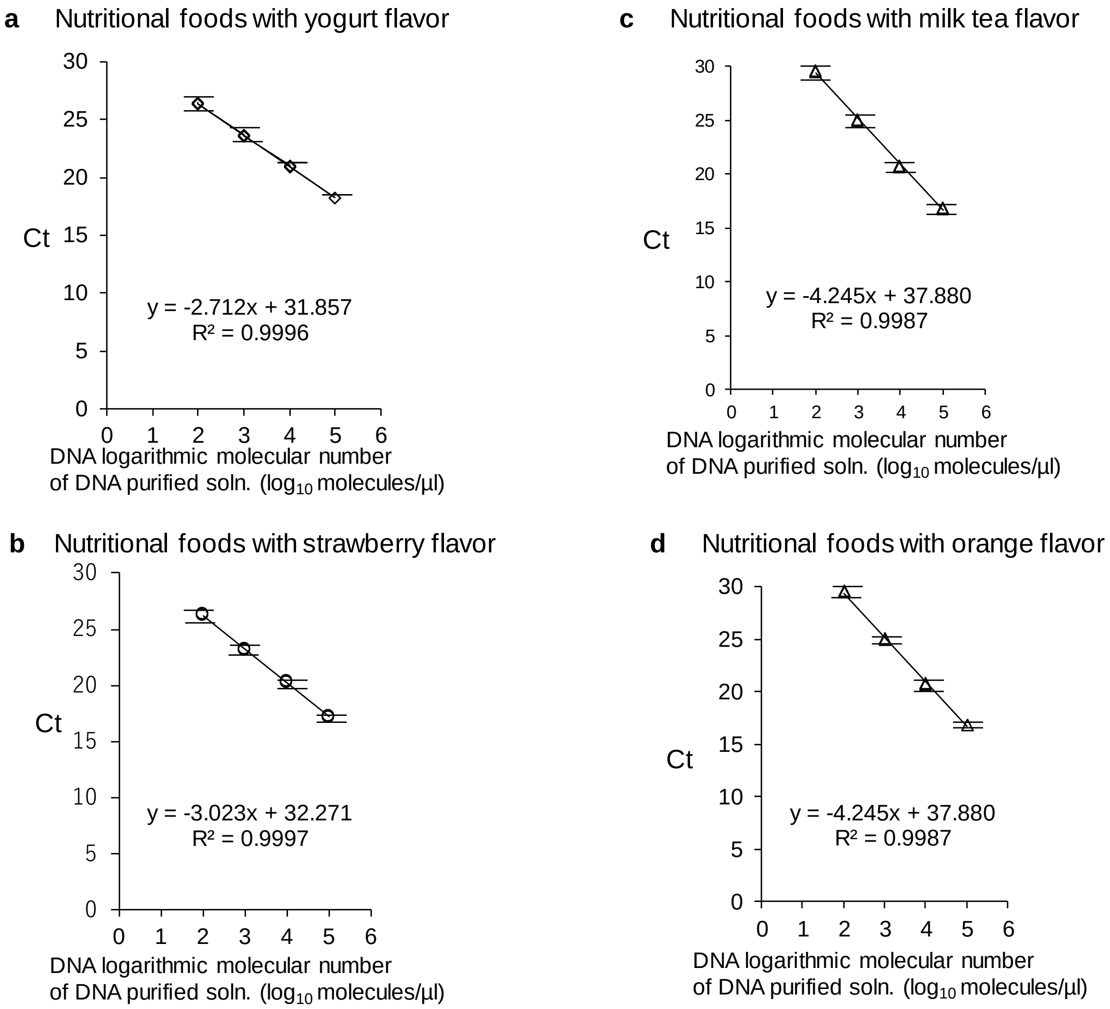

Supplementary Fig. S4. Measurement results of heat-killed *L. paracasei* standards using the matrices of DNA purified from 4 kinds of the relevant cell-free nutritional foods by the correlative qPCR.

DNA logarithmic molecular number of purified DNA solution (log10 molecule/μl) of the standards was set as X-axis, and then the correlative Ct values were plotted on Y-axis for standard curves. Standard curves in three replicates are presented as the mean (open diamond shape, ◊; open circle, ○; open triangle, Δ; closed cross, ✠) ± SD (error bar) with black color.

(**a**) A 10-fold diluted purified DNA solution stemming from the heat-killed *L. paracasei*-originally-supplemented product (NFY): Ct data (20.58, 20.26 and 20.90); the associated no-diluted (original) purified DNA solution: Ct data (17.73, 18.03 and 18.29).

(**b**) A 10-fold diluted purified DNA solution stemming from the heat-killed *L. paracasei*-originally-supplemented product (NFS): Ct data (21.07, 21.94 and 21.42); the associated no-diluted (original) purified DNA solution: Ct data (17.83, 17.98 and 18.61).

(**c**) A 10-fold diluted purified DNA solution stemming from the heat-killed *L. paracasei*-originally-supplemented product (NFM): Ct data (20.48, 20.40 and 20.62); the associated no-diluted (original) purified DNA solution: Ct data (17.71, 18.11 and 17.93).
(**d**) A 10-fold diluted purified DNA solution stemming from the heat-killed *L. paracasei*-originally-supplemented product (NFO): Ct data (21.44, 21.09 and 21.24); the associated no-diluted (original) purified DNA solution: Ct data (17.90, 18.26 and 17.68).

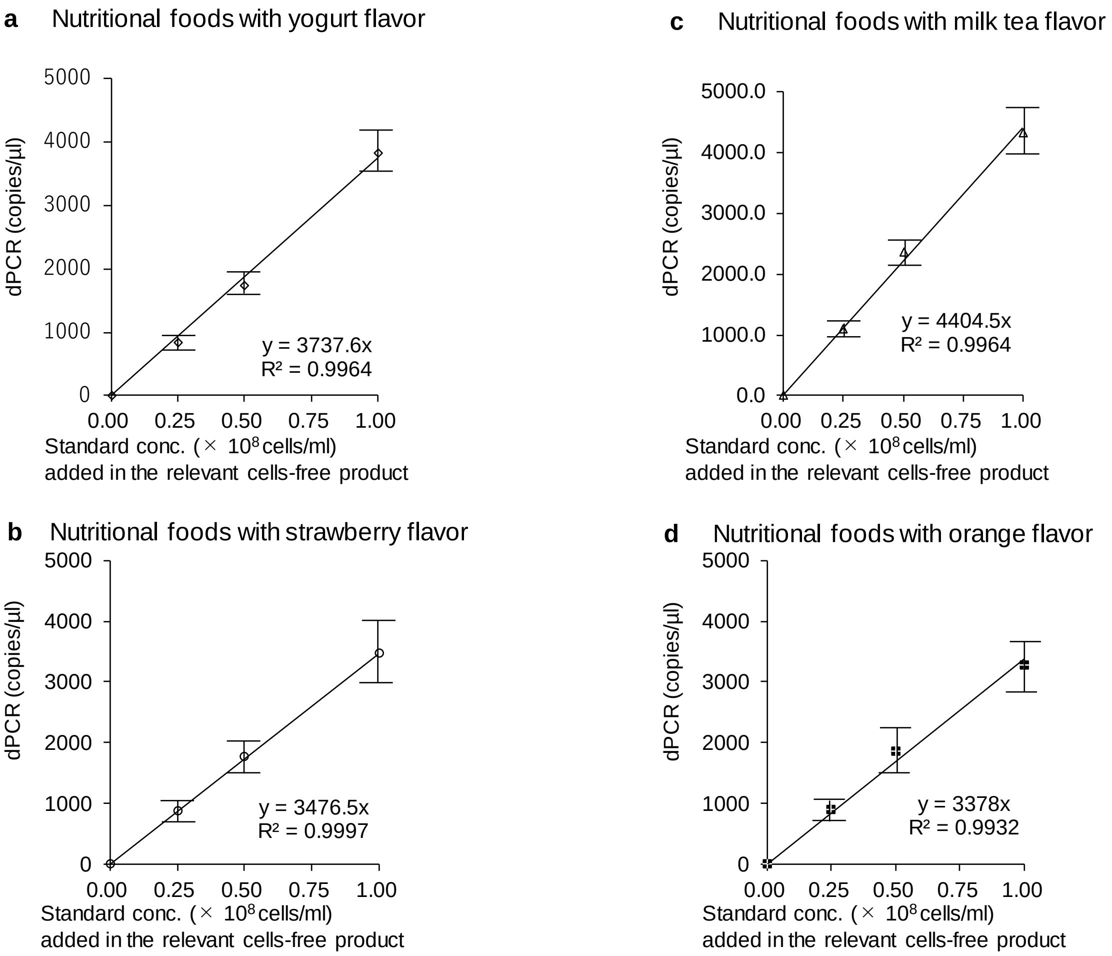

Supplementary Fig. S5. Measurement results of heat-killed *L. paracasei* standards using 4 kinds of the relevant cell-free nutritional foods by the correlative dPCR. Concentration with standards was set as X-axis, and then the correlative dPCR measurement values were plotted on Y-axis to obtain standard curves. Standard curves in three replicates are presented as the mean (open diamond shape, ◊; open circle, ○; open triangle, Δ; closed cross, +) ± SD (error bar) with black color.

(**a**) Three replicates of the dPCR measurement data for heat-killed *L. paracasei* originally supplemented NFY (nutritional foods with yogurt flavor) product were 3112.8, 3629.7 and 3914.5 copies/µl. (**b**) The associated 3 replicates for NFS (nutritional foods with strawberry flavor) product were 1915.6, 1948.9 and 2808.8 copies/µl. (**c**) The relevant 3 replicates for NFM (nutritional foods with milk tea flavor) product were 2706.5, 2834.2 and 3157.1 copies/µl. (**d**) The associated 3 replicates for NFO (nutritional foods with orange flavor) product were .
1983.7, 2174.9 and 2807.1 copies/µl.

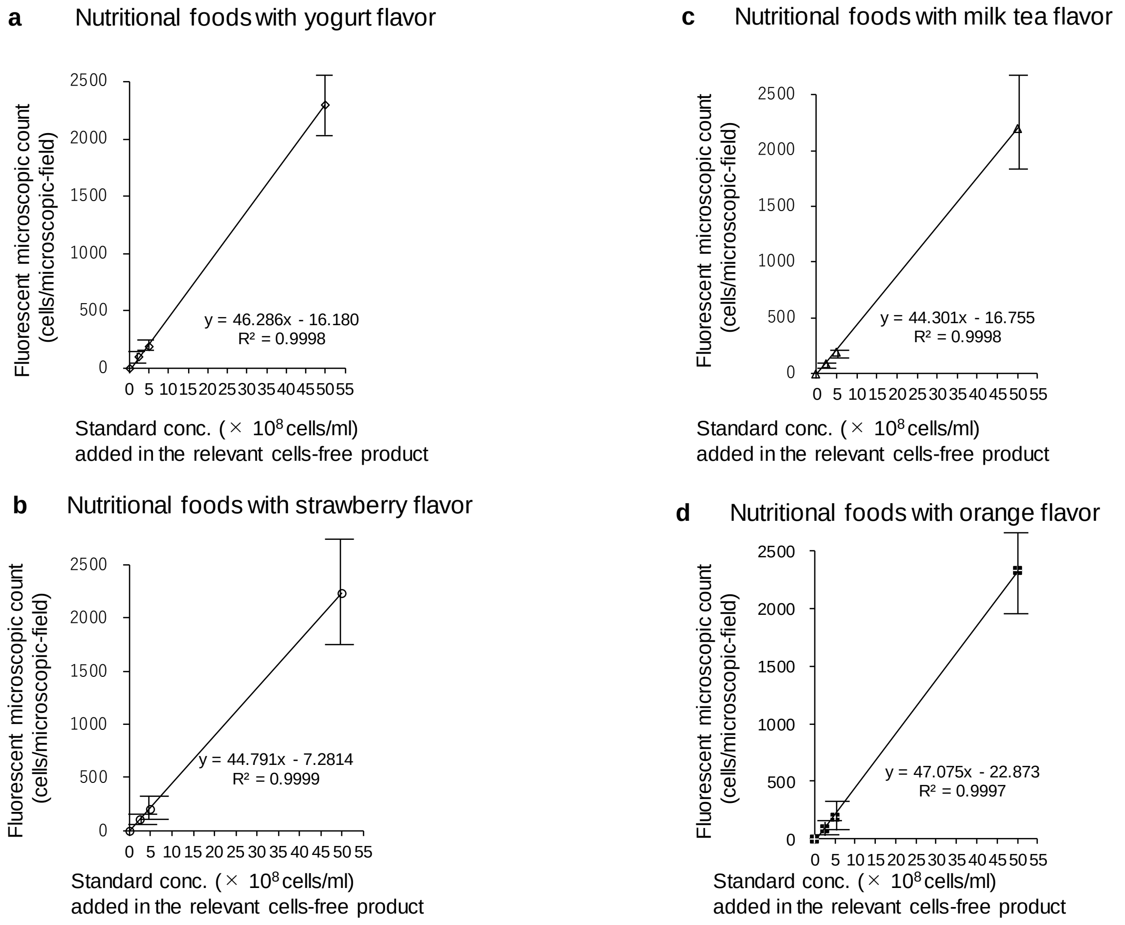

Supplementary Fig. S6. Measurement results of heat-killed *L. paracasei* standards using 4 kinds of the relevant cell-free nutritional foods by the fluorescent microscopy with PI staining.

Concentration with standards (×10^8 cells/ml) was set as X-axis, and then the number of the correlative fluorescent microscopic count were plotted on Y-axis for standard curves. Standard curves in three replicates are presented as the mean (open diamond shape, ◊; open circle, ○; open triangle, Δ; closed cross, +) ± SD (error bar) with black color.
(**a**) Three replicates of measurement data for heat-killed *L. paracasei* originally supplemented in NFY (nutritional foods with yogurt flavor) product were 50, 54 and 55 cells/microscopic-field. (**b**) The associated 3 replicates for NFS (nutrient foods with strawberry flavor) product were 33, 48 and 63 cells/microscopic-field. (**c**) The relevant 3 replicates for NFM (nutrient foods with milk tea flavor) product were 44, 54 and 61 cells/microscopic-field.
(**d**) The associated 3 replicates for NFO (nutrient foods with orange flavor) product were 39, 55 and 77.

References

1. Soejima, T. *et al*. Method to detect only live bacteria during PCR amplification. *J. Clin. Microbiol.* **46**, 2305-2313 (2008).
